# Categorial Compositionality III: F-(co)algebras and the Systematicity of Recursive Capacities in Human Cognition

**DOI:** 10.1371/journal.pone.0035028

**Published:** 2012-04-13

**Authors:** Steven Phillips, William H. Wilson

**Affiliations:** 1 Mathematical Neuroinformatics Group, Human Technology Research Institute, National Institute of Advanced Industrial Science and Technology, Tsukuba, Ibaraki, Japan; 2 School of Computer Science and Engineering, The University of New South Wales, Sydney, New South Wales, Australia; Indiana University, United States of America

## Abstract

Human cognitive capacity includes recursively definable concepts, which are prevalent in domains involving lists, numbers, and languages. Cognitive science currently lacks a satisfactory explanation for the systematic nature of such capacities (i.e., why the capacity for some recursive cognitive abilities–e.g., finding the smallest number in a list–implies the capacity for certain others–finding the largest number, given knowledge of number order). The category-theoretic constructs of initial F-algebra, catamorphism, and their duals, final coalgebra and anamorphism provide a formal, systematic treatment of recursion in computer science. Here, we use this formalism to explain the systematicity of recursive cognitive capacities without ad hoc assumptions (i.e., to the same explanatory standard used in our account of systematicity for non-recursive capacities). The presence of an initial algebra/final coalgebra explains systematicity because all recursive cognitive capacities, in the domain of interest, factor through (are composed of) the same component process. Moreover, this factorization is unique, hence no further (ad hoc) assumptions are required to establish the intrinsic connection between members of a group of systematically-related capacities. This formulation also provides a new perspective on the relationship between recursive cognitive capacities. In particular, the link between number and language does not depend on recursion, as such, but on the underlying functor on which the group of recursive capacities is based. Thus, many species (and infants) can employ recursive processes without having a full-blown capacity for number and language.

## Introduction

Many cognitive domains include recursively definable concepts (i.e., concepts defined with reference to themselves), such as domains involving lists, numbers, or languages. In card games, for example, a deck of cards can be defined (recursively) as a top card (perhaps turned face up to reveal its value) and a (remaining) deck of cards. To include finite decks, the definition has an alternative clause specifying an empty deck; that is, a deck is either empty, or contains a top card and a (smaller) deck. Operations on recursively defined concepts may also be defined recursively. For example, removing jokers from a deck of cards can be defined (recursively) as removing the top card if it is a joker and then removing jokers from the remaining deck of cards. Given that you don't find people who can remove the jokers from a hand of seven cards without being able to remove jokers from a deck of fifty-three, recursion-related capacities are further instances (see below) of the systematic nature of human cognition.

Systematicity is a property of human cognitive architecture (i.e., the basic processes and modes of composition that together afford cognition) whereby cognitive capacity is organized around groups of related abilities. A standard example since the original formulation of the problem [1] has been that you don't find people with the capacity to infer John as the lover from the statement *John loves Mary* without having the capacity to infer Mary as the lover from the related statement *Mary loves John*. In general, an instance of systematicity is when a cognizer has cognitive capacity 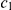 *if and only if* the cognizer has cognitive capacity 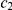 (see [2]). In this format, we say, e.g., that systematicity is evident where one has the capacity to remove the jokers if and only if one has the capacity to remove the aces (assuming, of course, one has the capacity to identify jokers and aces).

The classical explanation for systematicity has two components: (1) combinatorial syntactically structured representations; and (2) processes that are sensitive to (i.e., compatible with) those syntactic structures. In a classical cognitive architecture, mental representations of constituent entities (e.g., *John*, *Mary*) are tokened (instantiated) whenever the mental representations of their complex hosts (e.g., *John loves Mary*) are tokened, with the meaning of a complex host representation obtained (recursively) from the meaning assigned to its constituent mental representations and their syntactic relationships. By analogy to language, this form of mental representation is called a *language of thought* (LoT) [3].

The three aspects of systematicity, i.e., *systematicity of representation*, *systematicity of inference*, and *compositionality of representation* [1], can often be derived from classical cognitive architectures, because the same component processes are often used for each and every member of a group of systematically-related capacities. For instance, a classical system has the capacity to represent *John loves Mary* if and only if the system has a capacity to represent *Mary loves John* when the common component process is something like a production rule: 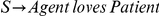 (where *John* and *Mary* can both be produced from *Agent* and *Patient* by other production rules)–systematicity of representation. Likewise, a classical system has the capacity to infer John as the lover in *John loves Mary* if and only if it has the capacity to infer Mary as the lover in *Mary loves John* given a common process that is sensitive to the syntactic structure whereby the lover constituent is represented by the first token–systematicity of inference. Also, the capacity to assign the semantic content of John being the lover of Mary to the representation *John loves Mary* if and only if there is the capacity to assign the semantic content of Mary being the lover of John to the representation *Mary loves John* derives from the tokening principle (above) mediating classical representations and processes: the process for juxtaposing tokens (symbols) *John*, *loves*, and *Mary* to form *John loves Mary* with corresponding semantic content is the same process that is used to form *Mary loves John* with corresponding content.

Classical compositionality would seem to provide an elegant explanation for systematicity with regard to recursive capacities, even though it fails to provide a full account of systematicity generally [4]. (Classical theory fails to provide a complete explanation because one can construct syntactically compositional systems that support some but not all members of a group of systematically-related cognitive capacities. Additional, so-called *ad hoc*, assumptions are needed to derive only those classical cognitive architectures that support systematicity–see [4] for an extensive and detailed analysis. This problem for classical theory echoes the one originally raised against connectionism as a *theory* of cognitive architecture [1], [5].) For recursive definitions, like the deck of cards, one self-referencing rule typically covers all cases (bar the terminating case, such as the empty deck). For example, removing jokers from a single hand, or an entire deck invokes the same component process. The two tasks only differ in the number of recursive steps.

### Classical compositionality without systematicity for recursion

However, the classical explanation with regard to recursive capacities still suffers the same general problem (illustrated below) that it suffers for non-recursive capacities. Suppose one card game requires removing the lowest value card in the hand dealt, while another card game requires removing the highest value card. In schema terms, given knowledge of the relative value of the cards, a person has the capacity to remove the lowest valued card if and only if a person has the capacity to remove the highest valued card, given that they know the relative values of each card. In everyday terms, you never come across card players who can play one of the games, but not the other. Classical theory admits at least two general schemes for realizing these capacities, recursive and non-recursive iteration, without requiring that they share a common component process. Hence, classical theory admits the case of having one capacity without having the other. Moreover, even under restriction to a single recursive (or, non-recursive) scheme, there remains an assumption that the processes for making inferences from representations of recursively-definable entities are *compatible* with the processes for building those representations (see [6]). For these reasons, classical theory does not provide a complete explanation of systematicity, even for recursively defined capacities.

To illustrate the problem just outlined, suppose the following recursive procedure, *lowest*, for identifying the lowest valued card in a deck of cards (containing at least one card):
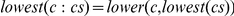


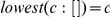
where a deck of cards 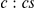 is represented by a recursively defined list with 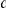 as the top card and 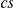 as the remaining deck, 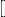 is the empty deck, and 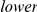 returns the lower of two cards. Suppose, also, the following classical non-recursive procedure, *highest*, for identifying the highest valued card:
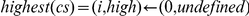


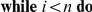


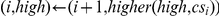


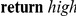
where deck 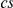 is represented by an array of 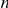 cards with position indexed by 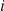 (i.e., 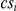 is the *i*th card), 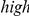 maintains a representation of the (currently) highest card, 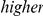 returns the higher of two cards (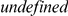 is some value guaranteed to be lower than any card), and 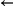 indicates variable-value assignment. Clearly, the two procedures do not share any component processes, and so do not provide a basis for systematicity, even though systematicity could be supported when both tasks are implemented in either the first style only, or the second style only. (In fact, entire programming languages have been designed to support only the first–e.g., Haskell–or only the second–e.g., Basic–style of recursion/iteration.) Notice that we are not unfairly stressing classical theory by apportioning capacity at the level of constituents–systematicity concerns “molecular”, not “atomic” capacities [1]. Rather, given constituent capacities *lower* and *higher*, classical theory admits two independent compositional forms, as the example illustrates. Notice, also, that even when confined to recursive or iterative style, there is still the assumption that the deck of cards is represented in a particular order. Item order is crucial for, say, recalling the first item. An architecture that constructs lists with one order, but accesses them assuming another will fail to exhibit systematicity. This further problem is an analog of the one highlighted for the classical explanation in regard to non-recursively defined entities [6].

### Category theory explanation of (non-)recursive systematicity: outline

An explanation for non-recursive systematicity without *ad hoc* assumptions was given in [6], [7], using a branch of mathematics called *category theory* [8]. Briefly (and informally), our category theory explanation supposes that building blocks of a (categorial) cognitive architecture are “universal constructions”. In effect, a universal construction guarantees that each and every morphism (cognitive process) in the category (cognitive domain) of interest factors through (is composed of) a universal arrow (common component process) in a unique way (without requiring additional assumptions). The *ad hoc* aspects of previous approaches to systematicity are avoided because uniqueness is a built-in part of universal constructions. In this paper, we extend our category theory explanation to recursive capacities using universal constructions called an *initial F-algebra* and a *final F-coalgebra*, which have been extensively developed in computer science as a theoretical basis for recursive computations [9]–[12]. Our previous work [6], [7] dealt with non-recursive domains using a kind of universal construction called *adjoint functors*–a *functor* is a way relating categories, which can be viewed as a way of constructing objects and morphisms from one category based on those in another. The current work uses *endofunctors*, which relate categories to themselves, hence their relevance to recursion: from an initial *F*-algebra on an endofunctor 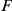 we get systematicity of inference; from the associated final *F*-coalgebra on the same endofunctor we get systematicity of representation; and from endofunctors composed of category-theoretic products we get compositionality of representation. In the next section (Methods), we introduce the category theory concepts needed for this explanation. Then, we present our explanation for systematicity in regard to recursively defined capacities, with specific examples (Results). Finally (Discussion), we provide some perspective on our explanation in terms of its potential limits, and some broader aspects of cognition, including integration with non-systematic capacities, and the debate over the relationship between recursion, number and language in humans and other species.

## Methods

Our approach to systematicity in recursive domains employs standard category theory constructs and methods that can be found in many general introductions to category theory (see, e.g., [8], [13]–[15]), and more detailed treatments of *F*-algebras and recursion (see, e.g., [16], [17]). A semi-formal presentation is provided here to facilitate an intuitive understanding of the background theory, with an expanded treatment provided in Text S1.

### Category

All category theory constructs “live” in a category of some description. Categories consist of objects and morphisms (or, maps) between them, satisfying certain conditions. A standard example is the category 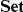, which has sets for objects and total functions between sets for morphisms. One way to think of a category in regard to cognition is as a cognitive (sub)system where the objects are (sets of) cognitive states and the morphisms are state-transforming cognitive processes.

#### Definition (Category, object, morphism, domain, codomain, composition)

A *category* 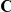 consists of a class of objects 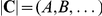; and for each pair of object 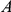, 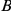 in 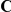, a set 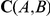 of morphisms (also called arrows, or maps) from 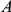 to 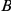 where each morphism 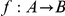 has 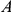 as its *domain* and 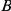 as its *codomain*, including the *identity* morphism 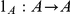 for each object 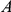; and a composition operation, denoted “
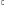
”, of morphisms 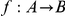 and , written 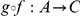 that satisfies the laws of:

- *identity*, where 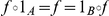, for all 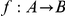; and
- *associativity*, where 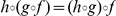, for all 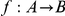, 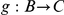 and 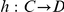.

For our purposes, we use the set-like category 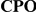 of *complete* partially ordered sets and *continuous* functions (see [15] for an introduction). As the term suggests, a complete partial order is a set with a partial order defined over it, plus some additional requirements (see Text S1). Though the technical details are important as part of a category-theoretic foundation for recursion, and thereby our explanation of systematicity, we omit the details here as they are not needed to convey the other concepts. Hence, for expository purposes, our examples use the category 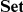. We refer to 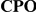 when being explicit about the category employed in our explanation for systematicity. Furthermore, in 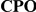, each object 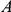 (except the empty set) includes a *least* element, denoted 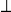, where 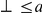 for all 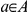, and each morphism 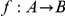 preserves this element, i.e., 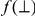 is the least element in 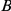. A least element is interpreted as the “undefined”, or “unknown” value. In cognitive terms, a system responds with unknown when given an unknown input. So, morphisms in 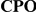 (Results section) are implicitly defined over these elements.

Certain objects and morphisms have special properties that warrant giving them names. In particular, an *initial* object is an object for which there is a morphism from it to every object in a category that has one; a *terminal* object is an object for which there is a morphism to it from every object in a category that has one. For example, in 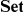 the initial object is the empty set, and a terminal object is any singleton (one-element) set. Initial and terminal objects are our first examples of universal constructs, and play an important role in our explanation of systematicity.

#### Definition (Initial object)

An *initial object* in a category 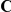 is an object, denoted 0, such that for every object 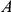 in 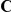 there exists a unique morphism 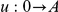 in 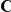.

#### Definition (Terminal object)

A *terminal object* in a category 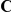 is an object, denoted 1, such that for every object 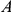 in 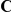 there exists a unique morphism 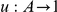 in 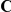.

Category theory employs a weaker, though more useful notion of “equality” called *isomorphism*. Two isomorphic constructs may be regarded as essentially the same, even though they are not identical. The notion of isomorphism commonly used in cognitive science derives from the mathematical versions of isomorphism, and the category theory notion of isomorphism is the most general of these.

#### Definition (Isomorphism)

A morphism 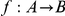 is an *isomorphism* if and only if there exists a morphism 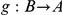, such that 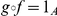 and 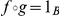. If 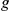 exists, then it is said to be the *inverse* of 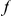 (also denoted 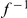). If 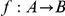 is an isomorphism, then 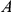 is said to be *isomorphic* to 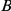, written 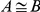.

Category theory also provides two basic, principled means of combining objects, called *product* and *coproduct*, where the prefix “co” is often used to label *dual* constructions, i.e., constructions obtained by reversing the directions of the morphisms of the other construct. One can think of a (co)product as a syntax-free notion of compositionality. Note that not all products and coproducts actually exist in all categories.

#### Definition (Product of objects)

A *product of objects* 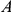 and 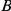 in category 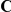 is, up to a unique isomorphism, an object 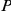 (also denoted 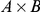) together with two morphisms (sometimes called *projections*) 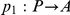 and 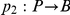, jointly expressed as 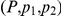, such that for every object 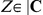 and pair of morphisms 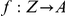 and 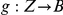 there exists a unique morphism 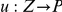, also denoted 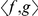, such that the following diagram commutes:
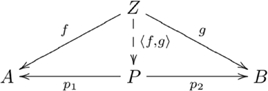
(1)


By a straightforward extension, the *finite product* of 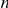 objects 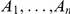 is 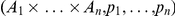. In the category 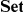, for example, the Cartesian product 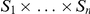 of 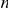 sets is a product of those sets in the categorical sense. The projections 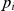, for 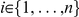, are the maps 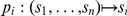. The symbol 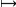 indicates a mapping of an element in a domain, so 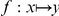 is the same as saying 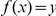.

(“Commute” means that any two paths, with at least one path composed of more than one morphism, with the same start object and the same end object are equal, e.g., 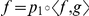, in Diagram 1.)

#### Definition (Coproduct of objects)

A *coproduct of objects* 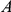 and 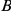 in category 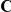 is, up to a unique isomorphism, an object 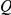 (also denoted 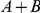) together with two morphisms 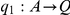 and 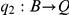, jointly expressed as 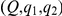, such that for every object 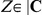 and pair of morphisms 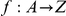 and 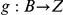 there exists a unique morphism 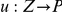, also denoted 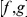, such that the following diagram commutes:
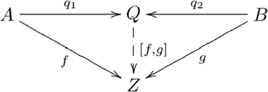
(2)


By a straightforward extension, the *finite coproduct* of 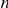 objects 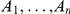 is 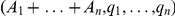.

### Functor

Functors provide a principled means for relating categories. In the context of categories as cognitive systems, functors provide a means for relating cognitive systems in a structurally consistent manner. One can think of a functor as a kind of function between categories that maps objects and morphisms in a way that preserves identities and compositions–ordinary functions only map objects, not morphisms. However, the requirement that identities and compositions are preserved means that not every such function is a functor.

#### Definition (Functor)

A *functor* 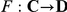 is a map from a category 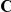 to a category 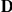 that associates each object 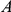 in 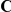 to an object 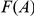 in 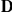; and each morphism 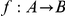 in 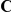 to a morphism 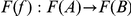 in 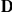, and is structure-preserving in that 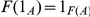 for each object 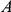 in 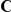, and 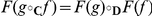 for all morphisms 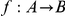 and 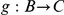.

One kind of functor is an *endofunctor* from a category to itself, hence its relevance to recursion.

#### Definition (Endofunctor)

An *endofunctor* 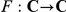 is a functor whose domain and codomain are the same category 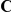.

An apparently trivial but actually useful example of an endofunctor is the *identity functor*, which maps every object and morphism to itself.

Other kinds of functors, such as *polynomial functors*, are also important for a categorical basis of recursion. The formal details are provided in Text S1. Intuitively, one can think of a polynomial functor by analogy to a polynomial function, but with the 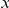 of a normal polynomial replaced by the identity functor, and the constants of a polynomial replaced by constant functors.

### *F*-Algebra

A category theory treatment of recursion starts with the concept of an *F-algebra* constructed on an endofunctor 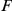. One can build up an intuition of *F*-algebras from the more familiar notion of elementary algebra. Elementary algebra consists of operators (e.g., negation, addition) that apply to and return numbers. The key difference is that *F*-algebra operators are defined in terms of endofunctors, affording recursion.

#### Definition (*F*-algebra)

For an endofunctor 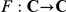, an *F-algebra* is a pair 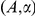, where 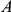 is an object and 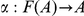 is a morphism in 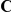. For an example, see Text S1.

#### Definition (*F*-algebra homomorphism)

An *F-algebra homomorphism* 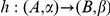 is a morphism 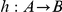 (in 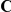) such that the following diagram commutes:
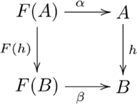
(3)That is, 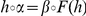.

#### Definition (Category of *F*-algebras)

For endofunctor 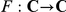, a category of *F*-algebras 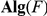 has *F*-algebras 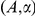 for objects, and *F*-algebra homomorphisms 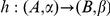 for morphisms.

An initial object in a category of *F*-algebras (if one exists) is called an *initial F-algebra*. And, just like an initial object in any other category (that has one), it is a universal construction: for every *F*-algebra in that category there exists a unique *F*-algebra homomorphism to it from an initial algebra, hence the importance of initial algebras to the systematicity of recursive capacities.

#### Definition (Initial algebra)

An *initial F-algebra* 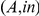, hereafter also simply called an *initial algebra*, is an initial object in the category of 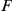-algebras 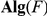. That is, there exists a unique *F*-algebra homomorphism from 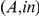 to every *F*-algebra in 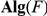.

#### Definition (Catamorphism)

A *catamorphism* 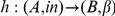 is the unique *F*-algebra homomorphism from initial *F*-algebra 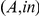 to *F*-algebra 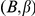. That is, 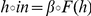, and the uniquely specified 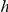 for each such 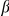 is denoted 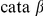 (i.e., 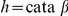), as indicated in the following diagram:
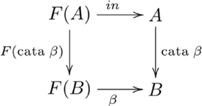
(4)(Catamorphisms are also denoted by so-called *banana brackets*, see [18].)

Duals: *F*-algebra, initial algebra, and catamorphism have dual constructs called *F-coalgebra*, *final coalgebra*, and *anamorphism* (respectively), which are also used in our explanation for systematicity. Details are provided in Text S1. Here, we just note that, like product and coproduct, they are related by reversal of the directions of the morphisms that are involved in their respective definitions.

### An initial F-algebra for lists

An initial algebra for lists provides our category theory basis for an explanation of systematicity with respect to list-related cognitive capacities, such as identifying the smallest or largest item. More formal details are provided in Text S1. For an intuitive understanding, recall (from the Introduction) our informal definition of a deck of cards as being a top card and a (remaining) deck of cards, or an empty deck. This definition is an instance of a list, which is a head element and a (remaining) list, or an empty list. So, list-related processing generally has two aspects: one for empty lists and one for processing non-empty lists, which consist of a head, and a remaining list (tail). In category theory terms, an initial algebra for constructing lists is the pair 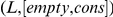, where 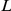 is a set of lists, and 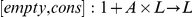 is the list-constructing morphism, consisting of the constant function 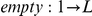 for constructing the empty list 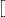, and the binary function 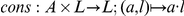 for constructing the list with element 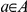 prepended () to list 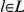. The function 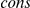 will be re-used later in the paper. If, for example, 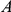 is the set of natural numbers 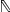, then 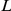 is the set of all finite natural number lists. Catamorphisms from this initial algebra to an *F*-algebra 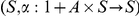 have the form of a recursive function 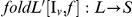, where 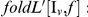


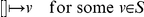


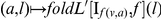
indicated in commutative diagram
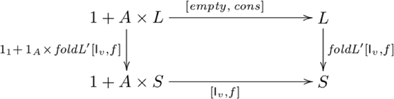
(5)


For instance, summing a list of numbers is 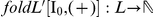: e.g., 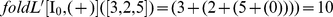. For a category theory development of various folds, see [17], and for corresponding concrete implementations in the Haskell programming language [19], see [20].

Notational convention: For morphisms, 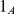 indicates the identity morphism on object 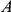, and 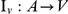 indicates a constant morphism (function) that maps all elements 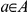 to the same element 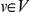. In Diagram 5, for example, a morphism 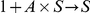 is automatically of the form 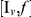 for some 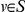 and some 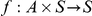. The names that we shall use for variants of *fold* include an object name that is the argument to the underlying functor. For example, *foldL* indicates a fold for the set of lists 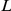. To reduce bracketing, we assume that product (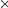) binds more tightly than (i.e., takes precedence over) coproduct (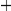), so e.g., 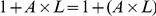, and arguments to *fold* bind more tightly than arguments to the resulting function, so e.g., 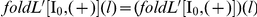. The prime (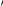) signifies folding from the end of the list (or analogous structure, see fold for numbers in Text S1), as opposed to folding from the front of the list (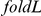, see Text S1). How this difference relates to systematicity is detailed in Text S1, and discussed in the last section.

### Universal constructions

Specific kinds of universal constructions were used to provide category theory explanations for the systematicity and quasi-systematicity of non-recursive relational structures in [6], [7]. Initial algebras (and final coalgebras) are another kind of universal construction that we use here to extend our explanation of systematicity to recursive capacities. Universal constructions (if they exist) in a category are characterized by a single *(co)universal morphism* which is a factor (via composition of morphisms) of all morphisms in the category, hence their relevance to systematicity: a (co)universal morphism underpins each and every group of systematically-related cognitive capacities. Initial algebras are instances of couniversal morphisms; final coalgebras are instances of universal morphisms (defined in Text S1).

#### Definition (Couniversal morphism)

Given an object 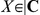 and a functor 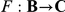, a *couniversal morphism* from 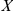 to 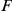 is a pair 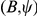 where 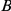 is an object of 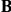, and 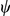 is a morphism in 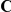, such that for every object 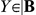 and every morphism 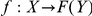, there exists a unique morphism 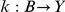, such that 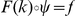, as indicated by commutative diagram
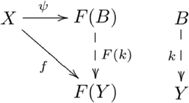
(6)


#### Definition (Universal construction)

A *universal construction* is either a universal morphism, or (its dual) a couniversal morphism.

## Results

Our explanation for systematicity proceeds in two stages: In the first stage, we use the universal property associated with initial algebras and catamorphisms to provide a category theory explanation for systematicity of inference in domains involving lists, numbers, and trees (relating to language). Systematicity of inference assumes processes for systematically constructing representations of the entities from which inference proceeds. The second stage of our explanation uses the dual notion of a final coalgebra to provide a corresponding category theory explanation for systematicity of representation. These two components of our explanation are necessarily connected, because the structure morphism 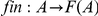 of the final coalgebra is the inverse of the structure morphism 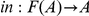 of the initial algebra for the functor underlying the category of algebras and coalgebras considered. The third aspect of systematicity, compositionality of representation, derives from endofunctors constructed from products. Since we have already shown that categorical products explain compositionality of representation [7], we do not repeat our explanation of this aspect of systematicity here. Then, we turn our attention to the relationships between these domains, and why number-, list-, and language-related capacities are not necessarily systematically related to each other. This result sheds light on why species and infants can have a capacity for recursion without having a capacity for language–systematic recursive capacity is tied to the underlying endofunctor; it is not a language-specific recursion construct–which we discuss in the next section.

### Systematicity: list-related capacities

Working with lists is a common, everyday cognitive activity, whether it be working through a shopping list, totaling money in hand, searching for a credit card, or entering an identification code. The explanation for this kind of systematicity is based on an initial algebra and associated catamorphisms in a category of *F*-algebras on a particular functor 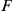.

#### Finding the smallest/largest item

We return to the example of systematicity raised (in the Introduction) as a problem for classical theory: a common task is to select the smallest or largest item in a collection of items. Systematicity, for this case, means that if one has the capacity to distinguish the relative sizes of items, and one has the capacity to identify the smallest item in a list of items, then one also has the capacity to identify the largest item in a list of items. Here, we illustrate our account of systematicity with respect to the natural numbers, i.e., where the type 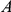, in the definition of catamorphism (in Diagram 5), is 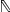. For other domains, such as selecting the ripest apple, largest watermelon, tallest player, etc., 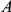 is set to the appropriate type for that domain. For current purposes, it suffices to set the fold of an empty list to infinity (i.e., from Diagram 5, 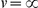), effectively meaning that the smallest number in any one-item list is that item. The function 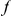 in Diagram 5 is 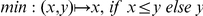, which returns the smaller of two items. So, the catamorphism for identifying the smallest number is 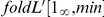, as indicated in commutative diagram
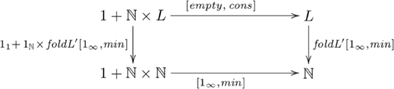
(7)


For example, 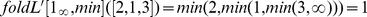. By replacing 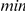 in Diagram 7 with 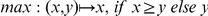, and 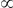 with 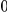 (or 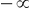 for lists of integers or reals), we have the catamorphism that corresponds to identifying the largest number. For example, 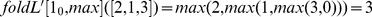. Since the two computations have the couniversal morphism 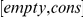 as the common component, this arrangement accounts for systematicity with respect to these capacities. Moreover, since the catamorphisms are uniquely determined, we have an account of systematicity without further (*ad hoc*) assumptions.

#### Accepting/rejecting/classifying items

A more general capacity is to select not just a single element from a list, but a sublist of elements satisfying some criterion. For example, when picking fruit one selects only the ripe ones based on some criterion of ripeness. When catching fish, one may reject those below a certain length. Or, when processing tomatoes, one may classify them on the basis of size. All such cases are examples of systematically related capacities: you don't find people who can identify the largest tomato without being able to classify them into small versus large. The initial algebra and catamorphisms that account for this form of systematicity are shown in commutative diagram
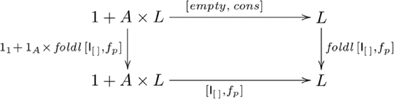
(8)where 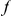 (in Diagram 5) is 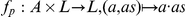, 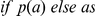, which is a function that accepts or rejects items depending on whether they satisfy condition 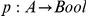, returning True when 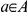 has the criterion property 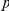, else False. In the case of classifying items, 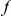 (in Diagram 5) is the function 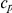, which returns a list of classes, one for each element, according to some classification criterion. That is, 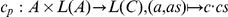, where 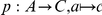 is a classification function, and lists are explicitly labeled with the type of their elements, e.g., 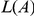 indicates a list of type 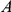. If we wish to group items from the original list into two lists on the basis of item classification, then 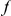 is the function 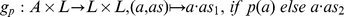, and 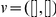, for the empty list, as indicated in commutative diagram
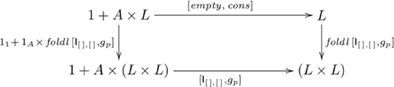
(9)where list type is not explicitly labeled, since all lists have the same type. For example, grouping a list of natural numbers into even and odd numbers is 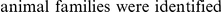, where 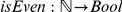, returns True if 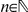 is even, otherwise False. Clearly, the capacity for classifying/grouping items can be generalized to more categories (e.g., small, medium, large). The explanation for systematicity without *ad hoc* assumptions parallels our explanation for systematicity in non-recursive domains [6], [7]: every capacity has as a factor the same couniversal morphism.

### Systematicity: number-related capacities

Number is another domain where humans exhibit systematicity over recursive capacities. Primary among these capacities are various forms of counting. *Simple counting* involves producing the sequence of numbers starting from a given number, such as counting the first ten numbers starting from one. Other forms of counting include *modular counting* (where the successor of a number may be 0, e.g., counting in 3's: 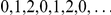), *stepwise counting* (such as counting in steps of two, or three, etc.), and *multiple counting* (where two or more counts are performed concurrently). In this section, we explain why systematicity with regard to these capacities follows from an *F*-algebra and catamorphism model without *ad hoc* assumptions. First, we present a category of *F*-algebras that includes number-related capacities, an initial algebra for this category and its associated general catamorphism. Then, we provide catamorphisms specialized to particular number-related capacities. Further details are given in Text S1.

#### Simple/stepwise/modular/multiple counting

The category of *F*-algebras that includes number-related capacities is constructed from the polynomial functor 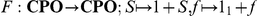. An initial algebra in this category is 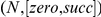, where 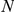 is a set used to model the natural numbers (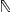), 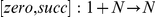, 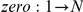 is a nullary function (equivalently, a constant) returning the element 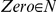, and 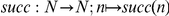 is a unary function returning the successor of element 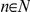. In this system, the number 2, for example, corresponds to 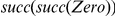. For this initial algebra, there is a general catamorphism called *foldN* (i.e., fold for numbers), defined as 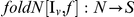, where
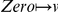


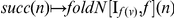


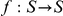
 is a unary function, and 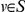 is a constant. The initial algebra and catamorphism are indicated in commutative diagram
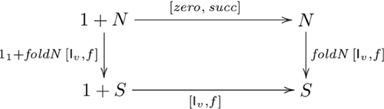
(10)


A simple counting task is to list out, in order, the first 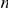 numbers starting from a given number 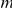: e.g., listing out the first five numbers from three yields the sequence: 3, 4, 5, 6, 7. The corresponding computation is an instance of the initial algebra and catamorphism given in the following commutative diagram:
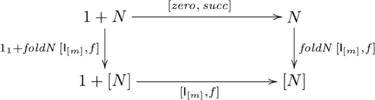
(11)where 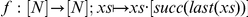, and 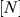 is the set of lists constructed from 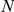,  is the list concatenation operator, and 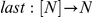 returns the last number 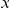 from a list of number 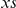. Thus, simple counting starting from 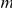 is the parameterized function 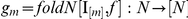, where 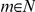.

Another simple counting task is to list out, in order, the first 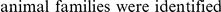 numbers from a given number 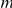 at intervals of 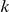: e.g., listing out the first four numbers from 1 at intervals of two yields the sequence: 1, 3, 5, 7. The corresponding diagram for this computation employs the same initial algebra and a unique catamorphism involving 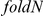 in Diagram 11, except that the function corresponding to 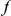 is now defined as: 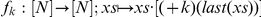, where the function 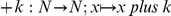, and 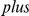 is a model for 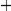 (see Text S1). Thus, *simple counting by interval* is the parameterized function 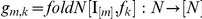.

These and other counting tasks (e.g., modular and multiple counting, see Text S1) involve the same initial algebra; i.e., the same couniversal morphism 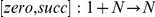, and a unique catamorphism involving 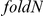. Hence, systematicity in regard to such capacities is explained by this universal construction, without further (*ad hoc*) assumptions. In cognitive terms, having the capacity for simple counting and knowing the interval relationships between numbers (e.g., 5 is two greater than 3) implies having the capacity to count in twos, because both capacities are uniquely composed of a common, universal component (namely 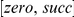). Thus, the presence or absence of this universal component is enough to imply the presence or absence of the entire collection of systematically-related capacities.

### Systematicity: language-related capacities

In this domain, we use an artificial grammar (for arithmetic expressions) to illustrate our explanation for systematicity with regard to language-related capacities. Artificial grammars are often used, because their forms are more easily adapted to the question at hand. We investigate a fragment of natural language (tail- versus center-embedded sentences), in the next section, in the context of capacities that may not be systematically related. Up to this point, we have addressed systematicity with respect to inference, e.g., why the capacity to infer the smallest list item is systematically related to the capacity to infer the largest list item–*systematicity of inference*. This aspect of systematicity assumes that the cognitive system also has the capacity to systematically represent the entities from which such inferences are made–*systematicity of representation*. Here, we also provide a category theory explanation for systematicity of representation, using the closely related, dual notion of an *F*-coalgebra.

#### Arithmetic expressions: systematicity of inference

The example in this section is based on [21], but adapted to model the *cognitive* capacity for evaluating numerical expressions. We first present a category of *F*-algebras that includes the language-related capacities of current interest, its initial algebra and associated general catamorphism, and then we specialize this catamorphism for arithmetic expressions (see Text S1, for further details).

The category of *F*-algebras that includes language-related capacities is constructed from the polynomial functor 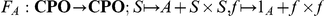. The *F*-algebras for the category 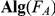 can be represented as pairs 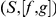, where 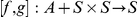, 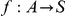 is a unary function, and 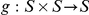 is a binary function. An initial algebra in this category is 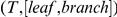, where 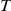 is the set of trees of type 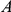, 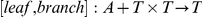, 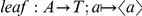 returns a tree consisting of a single leaf 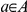, and 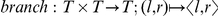 returns a tree consisting of a left branch 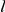 and a right branch 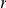, where 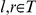. For example, a binary tree of numbers 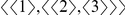 has a leaf 1 as its left branch, and a tree, with left leaf 2 and a right leaf 3, as its right branch. A catamorphism from initial algebra 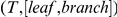 to an arbitrary *F*-algebra 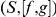 in 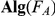 is the recursive function 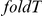 (i.e., fold for trees), defined as follows. The (higher-order) function 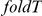 takes a unary function 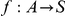 and a binary function 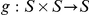 and returns the recursive function 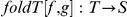, where
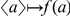


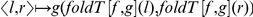
and 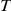 is a set of trees of type 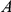, indicated in commutative diagram
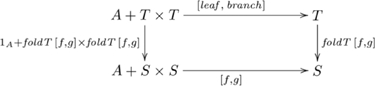
(12)


Suppose participants are given arithmetic expressions involving a particular operator, say, *addition*, e.g., 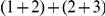, which they are required to evaluate. Given that participants can correctly evaluate such expressions, there is a host of other capacities that are also afforded provided that they have some other basic knowledge. For example, given knowledge of another binary operator, say, *subtraction*, participants can also evaluate the related expression 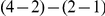 as 1. The specific catamorphism for the addition case is given in commutative diagram
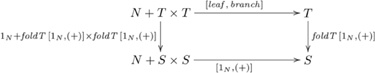
(13)


For the case of *subtraction*, the binary operator 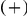 for addition is replaced with 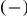 in Diagram 13. Hence, the second task is computed as 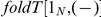. The universal construction common to these two capacities is the couniversal morphism 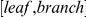. So, the explanation for systematicity is essentially the same as the explanations we provided for list- and number-related capacities, albeit based on a different underlying functor–the capacities for evaluating expressions involving addition and subtraction contain 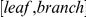 as the common factor.

The addition and subtraction examples only consider cases where each expression consists of only one kind of operator. A more developed ability is the capacity to evaluate expressions that include different operators, e.g., 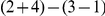. Such expressions require trees that explicitly include each operator, e.g., the tree 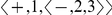 corresponds to the expression 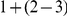. An initial algebra for such expressions is based on the functor 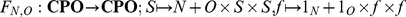. An initial algebra is 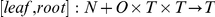, where 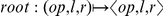, and 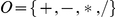 is the set of arithmetic operators. Here, the set of numbers 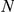 includes the reals. This example can be extended further by considering expressions that include operators of different arities, as in the expression 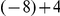. This extension requires yet another kind of tree algebra based on the functor 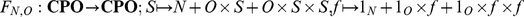. These possibilities raise the question of which tree to construct. Both systematicity of representation and the problem of determining which tree are addressed using the dual notion of an *F*-coalgebra, which we turn to next.

#### Arithmetic expressions: systematicity of representation

The previous section considered various systematically-related capacities for evaluating trees. These examples are instances of *systematicity of inference* [1], [4], [6]. Yet, such expressions are not given to the cognitive system in tree-form. Typically, such trees are assumed to be constructed from an input (list of characters) by another process. The input may take on several different formats: e.g., alpha-numeric, as in 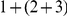, or word form, as in *one plus (two plus three)*, which correspond to the same tree. Again, these two forms are systematically related: one has the capacity to represent the expression 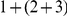 if and only if one has the capacity to represent the expression *one plus (two plus three)* assuming, of course, a person knows that *one*, *two* and *three* denote the same things as 1, 2 and 3 (respectively), and *plus* denotes the same thing as 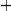. This form of systematicity is called *systematicity of representation* [1], [4], [6]. In this section, we show how systematicity of representation is addressed using coalgebras. Since a coalgebra on a functor 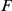 is intimately tied to its dual algebra on 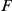, coalgebras also address the problem of determining which tree to construct.

Constructing trees from lists is achieved by a dual construction called an *F-coalgebra* [21] (see Text S1, for definitions). The explanation for systematicity in this case proceeds in a “dual” manner: i.e., every morphism in a category of *F*-coalgebras with a terminal (dual to initial) object, called a *final coalgebra* (dual to initial algebra) is composed of a unique *anamorphism* (dual to catamorphism) and a common final coalgebra. (Note the reversal in the order of composition compared with *F*-algebras.)

The development of the concept of final coalgebra derives from the dual definition of the concept of initial algebra, in this case in the category of *F*-algebras 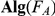 on the functor 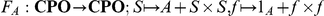. A final coalgebra in the dual category 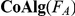 is 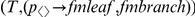, where conditional 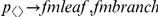 consists of a condition 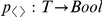 that tests whether 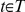 is a leaf (i.e., 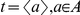), or a branch (i.e., 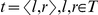), and associates functions 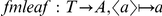, for retrieving a value from a leaf, and 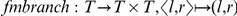, for retrieving a pair of left and right subtrees from a branch. There are more details on conditional functions in Text S1 and [9]. The dual category 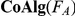 has *F*-coalgebras 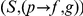 as objects, and *F*-coalgebra homomorphisms as morphisms. The anamorphism associated with this final coalgebra is called *unfoldT* (i.e., unfold for trees), defined recursively as 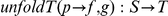


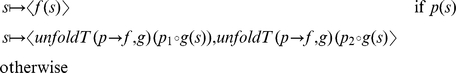
The final coalgebra and associated anamorphism are indicated in commutative diagram
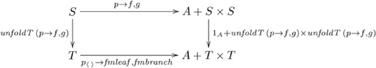
(14)


Diagram 14 indicates the general form of the anamorphism from which we need to specify a particular 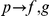 for our domain of arithmetic expressions. That is, we need to define the test function 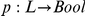, where 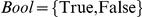 that determines whether an expression (i.e., list of characters, such as “1+(2+3)”) indicates a simple (value) or complex expression, and associated functions 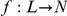 and 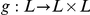 for transforming simple expressions into numbers and complex expressions into pairs of expressions, respectively.

Specifications of 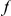 and 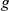 (in Diagram 14) are obtained from case analysis. Examples of simple expressions, which indicate values, are: “1”, “(2)”, and “((3))”, i.e., any well-formed expression that does not contain the “+” character. A complex expression is any well-formed expression that is not simple. So, 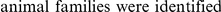 is the function 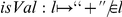 (and 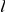 is a well-formed expression). Later, we show how this extends to other operators. Since 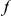 is associated with 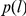 being true, we require a function to convert a string into a (internal) representation for the corresponding number, i.e., 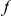 is the function 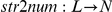 that converts a string of characters like “123” to the corresponding number 123. Finally, we need a function 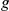 for complex expressions. Examples of complex expressions include: “1+2”, “1+(2+3)”, “(1+2)+3”, “(1+2)+(3+4)”, and so on. The purpose of 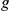 is to split an expression into two subexpressions, one corresponding to the left branch of the tree, and the other to the right branch. That is, 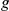 must split the expression at the topmost operator into two subexpressions containing the strings before and after the “+” symbol, after stripping off the outer brackets. Identifying the split point is also determined by case analysis: Basically, the split point is the first instance of “+” in the absence of an unmatched right bracket “)”. So, one simply maintains a counter, starting from 0 (i.e., no unmatched brackets, or top level), which is incremented/decremented on every occurrence of a left/right bracket, when read from left to right, and on finding a “+” when the counter is 0, splits the string at this point. For example, “(1+2)+3” is split into “(1+2)” and “3”. 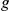 is this function 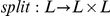. Thus, the function for parsing expressions into trees is the anamorphism 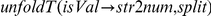.

Systematicity of representation (in this example, constructing trees) is obtained in the same way as systematicity of inference (*destructing* trees). Destruct is used in a technical sense as the dual to construct: e.g., to destruct a tree is to pull apart its constituents, which are either the left and right subtrees in the case of a branch, or the value of the leaf in the case of a leaf (see also Text S1). To represent the same tree from the expressions in word form, one simply replaces the argument 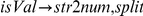 as appropriate. Thus, the function 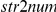 is replaced with 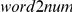 which converts numbers in word form (e.g., “one”, “two”, etc.) to their corresponding internal representation of number, and 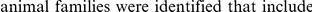 searches for the string “plus” rather than “+”. In any case, the resulting anamorphism factors through the same universal morphism, i.e., 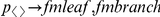 from Diagram 14.

Given initial algebra 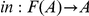 in a category 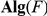, the corresponding final coalgebra 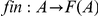 is guaranteed to exist, because 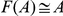, and indeed 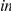 has as inverse 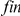. Thus, further (*ad hoc*) assumptions are not required to guarantee a correspondence between expressions and evaluations since they are indivisibly bound by the initial algebra/final coalgebra. By contrast, classical theory assumes that the processes for constructing syntactically compositional representations and the processes for systematically transforming those representations correspond [6]. Naturally, this result extends to other kinds of (final) initial (co)algebras, such as those pertaining to lists (see Text S1, for further details).

### Numbers, lists, and languages: are they systematically related?

The short answer is: *No*. A more technical explanation is provided in Text S1. Here, we simply point to the differences between the respective *F*-algebra categories, which are made obvious from the commutative diagrams for the initial algebras in each category. The basic point is that although tree-related capacities subsume list-related capacities, which in turn subsume number-related capacities (because, e.g., numbers can be represented as lists of 1s–in effect tally marks) the converse is not true: having a capacity for number does not in general afford a capacity for lists, which in turn does not in general afford a capacity for trees.

Notice from the commutative diagrams indicating initial algebras for number (Diagram 10), list (Diagram 5), and tree (Diagram 12) that the underlying endofunctor has a different form. The endofunctor underlying the *F*-algebra category including number, i.e., 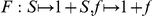 is an unparameterized polynomial functor of order one (cf. polynomial functions). For lists, the endofunctor is the parameterized polynomial functor of order one, 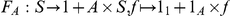, or equivalently, binomial functor 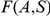. For language-related trees, the endofunctor is the parameterized polynomial functor of order two, 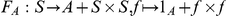, or the functor 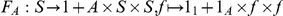. So, although a tree can be used to model a list, and a list can be used to model a number, generally, a number cannot be used to model a list, and a list cannot be used to model a tree in any *natural* way. Technically, that is to say, the three endofunctors are not related by natural equivalences (see Text S1). Thus, the forms of recursion that afford systematic cognitive capacity with regard to number do not afford systematic cognitive capacity with regard to list, nor tree, and likewise systematic capacity with regard to list does not afford systematic capacity with regard to tree.

#### Natural language: Tail- versus center-embedded recursion

A caveat to the distinction between number, list, and tree involves tail- versus center-embedded recursive constructions that are found in natural languages. The following example is taken from [22]. An example of a tail-embedded construction is *This is the cat that killed the rat that ate the malt that lay in the house that Jack built*. This expression in center-embedded form is *The malt that the rat that the cat killed ate lay in the house that Jack built*. In general form, we have production rules 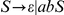 for tail-embedded sentences, and 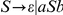 for center-embedded sentences, where 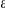 is the symbol for the empty string. Such constructions are indicative of the difference between regular grammars and context-free grammars. However, from the perspective of *F*-algebras, both are realized by list-related functors, albeit of different forms. The tail-embedded case is included in the category of *F*-algebras on the functor 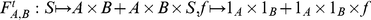, which includes the initial *F*-algebra for constructing lists such as 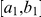, 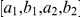, etc. The center-embedded case is included in the category of *F*-algebras on the functor 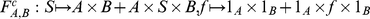, which includes the initial *F*-algebra for constructing lists such as 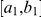, 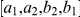, etc. However, these two functors are related by a natural isomorphism, suggesting that they are systematically related. We discuss the implications of this commonality in the next section.

## Discussion

Our explanation for systematicity with regard to recursive domains employs the same general category theory construct–universal construction–as our previous explanations for (quasi-)systematicity in regard to non-recursive domains [6], [7], albeit with different kinds of functors: here, for recursive domains, the universal constructions involved endofunctors (i.e., where the domain and codomain are the same category), whereas for non-recursive domains, the universal constructions involved adjoint functors (which are reciprocating, though not necessarily inverse, functorial maps between categories that are not necessarily the same. Every composition of left and right adjoints is an endofunctor, but not every endofunctor can be decomposed into a pair of adjoint functors. So, having some (primitive) form of systematicity over a recursive domain does not imply having systematicity for non-recursive domains. Nor, for that matter, does having the systematicity property for one recursive domain (e.g., numbers) imply the having the systematicity property for another recursive domain (e.g., lists), when the universal constructions involve functors not related by a natural isomorphism (this distinction also applies to non-recursive domains). See any of [8], [13], [15], [16] or Text S1 for a technical description of the concept of naturality. This functorial distinction between universal constructions has important implications for comparative and developmental psychology, which we discuss later.

This category theory explanation goes significantly beyond the classical one, despite some similarity between the two. The similarity between the two explanations lies in the use of common subprocesses underlying each and every member of a group of systematically-related cognitive capacities, which was the case in our explanation for non-recursive systematic capacities [6], [7]. In the case of recursive systematic capacities, the capacities are also intrinsically connected by two common subprocesses that are necessarily inverses of each other. Where we also go beyond the classical explanation is by introducing a principled distinction between commonalities that are universal (universal constructions), and those that are not (i.e., not necessarily universal). Note that we are not providing an “explanation” of systematicity by simply (re)defining it in the terms of some alternative, formal language. The formal concept of a universal construction has precise, empirically testable implications, a general schema for which we provided in Text S2. Hence, we can test the limits of systematicity, and thereby the limits of our theoretical explanation. Thus, although it may not seem obvious that adding a list of numbers is closely related to finding the smallest number (both involve a fold over lists), such cases can be put to an empirical test.

Our explanation for systematicity is based on universal constructions, but we require that the universal constructions arise from functors that are related by natural transformations, as we did previously: natural transformations were part of our explanation for non-recursive capacities in the form of universal constructions that are adjunctions–every adjunction consists of two natural transformations, and a collection of (co)universal morphisms.

### Limitations

There are two points at which our theory is likely to be incomplete: one point is where competence meets performance, such as when supposed systematically related capacities span memory or cognitive complexity limits (see [23] for a review and discussion of both kinds of limits). The other point is where systematic cognition meets non-systematic cognition: not all cognition is regarded as systematic; idioms (e.g., *John kicked the bucket*–i.e., he died–is not systematic with *Mary kicked the bucket [with her foot]*) are an example [1]. We discuss our theory in the context of both cases.

An example of the first point (competence versus performance) is the case of lists where the morphism 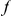 is not associative (e.g., subtraction): computing with a right-fold version of list fold means keeping all list items in memory (if presented once only), so systematicity would not extend beyond lists of more than a few items. Such cases are generally not regarded as evidence against the systematicity property–human cognition is *ceteris paribus* (e.g., memory requirements being the same) largely systematic (see [2]). Nonetheless, a more complete theory will address both aspects of cognition. Category theory may also provide independent principles for performance, since cognitive development-related limits in children were identified with the arity of the (co)product underlying the task [24]: e.g., the ability of children older than the median age of five years to perform transitive inference and class inclusion in the more difficult–cognitively complex–condition versus children younger than five was related to (co)product arity, i.e., binary versus unary (co)products. Note that here, too, the difference in “complexity” of the endofunctors for number (no/unary product of functors), list (binary product of constant and identity functors) and tree (binary product of two identity functors, or ternary product of constant and two identity functors). Product arity does not appear to distinguish the endofunctors underlying center-embedded versus tail-embedded recursion (their underlying functors are related by a natural isomorphism), yet center-embedded recursion is generally more difficult than tail-embedded recursion and appears to be unique to humans [22]. However, center-embedded recursion requires keeping all unmatched items in memory, so in expanded form center-embedded recursion employs a higher arity product. Nonetheless, performance (resource) related differences are beyond the scope of our theory as it currently stands.

In regard to the second point (systematic versus non-systematic cognition), category theory also provides a principled means for joining two cognitive (sub)systems via (co)products of categories (see Text S1, for a (co)product of categories definition), where one category models systematic cognitive capacity and the other non-systematic capacity, and (say) the coproduct category models both. An example of integrating systems with products is a hybrid distributive-symbolic model of grammar [25], where one category employs symbols and the other vectors. However, as Aizawa [4] explains, the required explanatory standard for hybrid theories is higher, because one must also explain why/when component theories are invoked. One possible reason is efficiency. Recall that a primitive form of addition was supported (systematically) by the category of *F*-algebras that included number-related capacities via 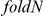, where the number of iterations was proportional to the size of the addends. The time required to add numbers can be reduced (and so efficiency increased) by memorizing the addition table for small numbers, which is what children are taught to do. However, addition via memorized associations is not a systematic process: one can memorize part of a table without memorizing the other part (this example is an analog of the phrase-book example in language [1]). So, utility may drive the cognitive system to employ a faster, though non-systematic process. However, utility is also outside the scope of our current theory. To meet this raised explanatory standard, one must explain why and under what conditions either component is employed, without resorting to *ad hoc* assumptions.

These sorts of questions can be put to an empirical test using the general schema for (non-)recursive systematicity detailed in Text S2. The basic format of this schema says that if participants have the capacity for the (co)universal component, and its composition with task specific components, then it must have the capacity for other tasks composed of the same (co)universal component. Success on a new task instance, i.e., without further feedback regarding the correct response, is an empirical test of systematicity.

### Perspective

At the core of our category theory explanation for systematic recursive capacity is a special pair of dual constructions: an (final) initial (co)algebra in a category of (co)algebras on a polynomial functor 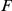. Although one can reverse the direction of any collection of arrows to form a dual, such duals may not exist in the category of interest (e.g., the existence of products in some category does not automatically guarantee the existence of coproducts in the same category). Yet, for categories of (co)algebras on a polynomial functor (final) initial (co)algebras are guaranteed to exist [16], and an initial algebra 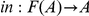 is guaranteed to have an inverse 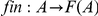, because the component objects are isomorphic (i.e., 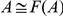), which constitutes a final coalgebra for the domains we have investigated. For polynomial functors, in general, an initial algebra (final coalgebra) is given uniquely by a final coalgebra (initial algebra), see [21]. So, the systematic relationship between representation and inference is guaranteed without further (*ad hoc*) assumptions, in contrast to the classical explanation where the link between the two is just assumed [6]. Notice, moreover, that this dual relationship between systematicity of representation and systematicity of inference is more general (and more useful) than an inverse. In the arithmetic expressions example, lists were represented as trees (systematicity of representation), but trees were evaluated as numbers (systematicity of inference). This form of duality goes beyond the simple inverse relationship between sentence recognition and generation found in parsing/production rules in a classical approach to language.

The capacity for recursion has been a contentious issue in the broader interests of cognitive science, which includes comparative and developmental psychology. Some argue that recursion is specific to humans and depends on language [26]; more particularly, a fully inductive (recursive) basis for number is specific to adults and distinct from infants' non-inductive basis [27]. In contrast, others claim a human language-like capacity for recursion in songbirds [28] (but, see [22]), and that adult understanding of number (in its fully induced form) is founded on a more primitive infant conception [29]. See also [30], for a review of the debate over the link between number and language. Our category theory treatment of recursive cognitive capacities provides a different perspective on this issue: specifically, as we have shown, the particular systematic capacities for recursion depend on the underlying functor, not a general capacity for recursion, as such. In particular, one can have a basic recursive capacity for number without having a full-blown capacity for language, because the functor underlying recursive number-related capacities does not provide a systematic basis for recursive language-related capacities, though by our account language-related recursive capacities afford number-related recursive capacities. Analysis of the songbird evidence [28] for supposed center-embedded recursion suggested that these birds were using a simple *counting* strategy [22], which accords with our *F*-(co)algebraic basis for recursion in cognition, where simple counting involves a fold for numbers, not lists or trees. Thus, other species (and infants) can have elementary recursive capacities without implying a full-blown capacity for number and language as they are available in adult humans.

The development of category-theoretic approaches to recursion in programming language design and automated refinement would seem to have little to do with a theory of cognitive architecture. Why, then, would one want to consider it as an approach to systematicity? In fact, the importance of the systematicity property to cognitive biological systems parallels the importance of abstraction in software systems engineering. Universal constructions in software design afford both economy of coding, and robustness: every call to an existing function obviates the need to write further code, and guarantees “correctness” across the various instantiations. That is, the same lines of code when called are guaranteed to work the same way every time; by contrast, any new line of code is “likely to introduce a new bug 50% of the time” (programmers' folklore). So, although the applications differ, the underlying principle is the same, and one can envisage evolution favouring the emergence of systematic processes because of the reproductive advantage afforded by this kind of efficiency. For this reason, category-theoretic approaches, which have worked well in theoretical computer science, are appropriate as an approach to the systematicity problem.

The goals of the cognitive and computer scientist are not entirely the same, of course. One potential point of divergence is with the origins of the structures underlying (cognitive) computation: computer scientists specify computational structures and identify their properties, whereas cognitive scientists are also concerned with their development/acquisition. Earlier categorical computational work focussed more heavily on *F*-algebras, while *F*-coalgebras were relatively underdeveloped [31]. For the systematicity problem, we see both as equally important, and their intrinsic connection suggests that their (co)habitation is important for a more complete theory of cognitive architecture. Just as the needs of computer scientists spurred the further development of *F*-(co)algebras for computing, the needs of cognitive scientists may spur yet further category theory development in this area. One area for future development is an account of how the universal constructions that we have proposed in our explanation for systematicity are modeled (implemented) by neural systems.

The classicist's approach to cognitive architecture is fundamentally limited not in advocating syntax, but in placing syntax at the foundation of their theory. Given the often *ad hoc* and idiosyncratic choices that go into programming language design, computer scientists in recent decades have turned to category theory for a deeper syntax-free understanding of the principles of computation. Cognitive science, as couched within the framework of computationalism, can likewise do better than lay foundations on the shifting sands of syntax.

We have adapted category theory principles for the beginnings of a categorial computational theory of cognitive architecture. Yet, if the answer to the systematicity problem is universal constructions, then the question that follows is, *How do the processes corresponding to universal constructions arise in the evolution/development of minds?* Perhaps, here too, category theory will provide an answer.

## Supporting Information

Text S1 **Further explanation of background category theory concepts used to explain systematicity.**
(PDF)

Text S2 **A category theory schema for testing systematicity.**
(PDF)

## References

[1] Fodor JA, Pylyshyn ZW (1988). Connectionism and cognitive architecture: A critical analysis.. Cognition.

[2] McLaughlin BP (2009). Systematicity redux.. Synthese.

[3] Fodor JA (1975). The language of thought. Language and Thought.

[4] Aizawa K (2003). The systematicity arguments. Studies in Mind and Brain.

[5] Fodor JA, McLaughlin BP (1990). Connectionism and the problem of systematicity: Why Smolensky's solution doesn't work.. Cognition.

[6] Phillips S, Wilson WH (2011). Categorial compositionality II: Universal constructions and a general theory of (quasi-)systematicity in human cognition.. PLoS Comput Biol.

[7] Phillips S, Wilson WH (2010). Categorial compositionality: A category theory explanation for the systematicity of human cognition.. PLoS Comput Biol.

[8] Mac Lane S (2000). Categories for the working mathematician. Graduate Texts in Mathematics.

[9] Bird R, Broy M (1987). An introduction to the theory of lists.. Logic of programming and calculi of discrete design.

[10] Hutton G (1999). A tutorial on the universality and expressiveness of fold.. J Funct Program.

[11] Malcolm G (1990). Data structures and program transformation.. Sci Comput Program.

[12] Spivey M (1989). A categorical approach to the theory of lists, volume 375 of *Lecture notes in computer science*..

[13] Awodey S (2006). Category theory. Oxford Logic Guides.

[14] Lawvere FW, Schanuel SH (1997). Conceptual mathematics: A first introduction to categories. Foundations of Computing.

[15] Pierce BC (1991). Basic category theory for computer scientists. Foundations of Computing.

[16] Manes EG, Arbib MA (1986). Algebraic approaches to program semantics.

[17] Bird R, de Moor O (1997). Algebra of programming.

[18] Meijer E, Fokkinga M, Paterson R (1991). Functional programming with bananas, lenses, envelopes and barbed wire.

[19] Jones SP (2003). Haskell 98 language and libraries: the revised report.

[20] Bird R (1998). Introduction to functional programming using Haskell. Prentice Hall Series in Computer Science.

[21] Hutton G (1998). Fold and unfold for program semantics.. Proceedings of the 3rd ACM SIGPLAN International Conference on Functional Programming.

[22] Corballis MC (2007). Recursion, language, and starlings.. Cognitive Sci.

[23] Halford GS, Cowan N, Andrews G (2007). Separating cognitive capacity from knowledge: a new hypothesis.. Trend Cogn Sci.

[24] Phillips S, Wilson WH, Halford GS (2009). What do Transitive Inference and Class Inclusion have in common? Categorical (co)products and cognitive development.. PLoS Comput Biol.

[25] Clark S, Coecke B, Sadrzadeh M, Bruza P, Lawless W, van Rijsbergen K, Sofge D, Coecke B (2008). A compositional distributional model of meaning.. Proceedings of the Second Symposium on Quantum Interaction.

[26] Hauser MD, Chomsky N, Fitch WT (2002). The faculty of language: what is it, who has it, and how did it evolve?. Science.

[27] Rips LJ, Bloomfield A, Asmuth J (2008). From numerical concepts to concepts of number.. Behav Brain Sci.

[28] Gentner TQ, Fenn KM, Margoliash D, Nusbaum HC (2006). Recursive syntactic pattern learning by songbirds.. Nature.

[29] Carey S (2009). The origins of concepts. Oxford Series in Cognitive Development.

[30] Gelman R, Butterworth B (2005). Number and language: how are they related?. Trend Cogn Sci.

[31] Gibbons J, Jones G (1998). The under-appreciated unfold.. Proceedings of the 3rd International Conference on Functional Programming.

