# Supplementary material for: Categorial Compositionality III: F-(co)algebras and the Systematicity of Recursive Capacities in Human Cognition
Source: PLoS One. 2012 Apr 13;7(4):e35028. doi: 10.1371/journal.pone.0035028 (PMC3325926; doi:10.1371/journal.pone.0035028)
Supplement: Text S1 — Further explanation of background category theory concepts used to explain systematicity. (PDF) [file pone.0035028.s001.pdf]

Text S1

This text provides additional definitions and explanations to help clarify the ideas presented in the main text, for readers unfamiliar with a category theory approach. We use standard category theory constructions and methods that can be found in general introductions to category theory (see, e.g., [1,2]), and introductions tailored for a categorical treatment of recursion (see [3–5]). The value added by this account of the material lies in the fact that it is self-contained, and contains only what is relevant to our applications of these concepts. Proofs of some well-known basic category theory results are included, as they help convey an understanding of our category-theoretic explanation of systematicity. For convenience, definitions that appear in the main text have been reproduced here.

Contents

Background **2**

Categories . . . 2

Duals . . . 4

Isomorphisms . . . 5

(Co)Products . . . 6

Functors . . . 8

Natural transformations . . . 11

***F*-algebras, initial algebras, and catamorphisms** **11**

Numbers . . . 14

Lists . . . 17

Trees . . . 24

Relation between numbers, lists, and trees . . . 27

Numbers from lists . . . 27

Lists from trees . . . 29

Numbers from trees . . . 31

No natural equivalences . . . 31

F-coalgebras, final algebras, and anamorphisms **32**

Distributive categories and conditional functions 33

Lists 34

Trees 39

Universal constructions **42**

(Co)Universal morphisms 42

Adjunctions 43

Background

In this section, we provide the background category theory concepts on which the concept of an F-algebra and universal construction are based.

Categories

Definition (Category, object, morphism, domain, codomain, composition). A *category* $\mathbf{C}$ consists of a class of objects $|\mathbf{C}| = (A, B, \dots)$; and for each pair of object A, B in $\mathbf{C}$, a set $\mathbf{C}(A, B)$ of morphisms (also called arrows, or maps) from A to B where each morphism $f : A \to B$ has A as its *domain* and B as its *codomain*, including the *identity* morphism $1_A : A \to A$ for each object A; and a composition operation, denoted "$\circ$", of morphisms $f : A \to B$ and $g : B \to C$, written $g \circ f : A \to C$ that satisfies the laws of:

- *identity*, where $f \circ 1_A = f = 1_B \circ f$, for all $f : A \to B$; and
- *associativity*, where $h \circ (g \circ f) = (h \circ g) \circ f$, for all $f : A \to B$, $g : B \to C$ and $h : C \to D$.

We briefly describe several relevant categories, then provide the definition of the category we use as the basis for our explanation of systematicity. A common first example of a category is **Set** whose objects are sets, and morphisms are total functions. Another, set-like, category is **Pos**, the category of *partially ordered* sets $(P, \leq)$, called *posets*, and *monotone* functions. A poset is a set P together with a binary relation $\leq$ defined on P that is reflexive ($a \leq a$), transitive ($x \leq y$ and $y \leq z$ implies $x \leq z$), and antisymmetric ($x \leq y$ and $y \leq x$ implies $x = y$). A monotone function $f : A \to B$ is *order-preserving*, i.e., $x \leq y$ implies $f(x) \leq f(y)$. For our purposes, we work in the category **CPO** of *complete pointed partial*

ordered sets and *continuous* functions (see [6] for an introduction, and [7] in the context of functional programming).

Definition (CPO). Let $(P, \leq)$ be a poset and S any subset of P. An *upper bound* on S is an element $x \in P$ such that for all $s \in S$ $s \leq x$. The set of upper bounds for S is denoted $UB(S)$, and $UB(\varnothing) = P$. The least upper bound, if it exists, is the least element of $UB(S)$, denoted $LUB(S)$. A poset $(P, \leq)$ is called *complete* if for every subset $S \subseteq P$ there exists a least upper bound $LUB(S)$. A poset $(P, \leq)$ is called *pointed* if there exists a least element, denoted $\perp \in P$, such that $\perp \leq p$ for all $p \in P$. An ω-*sequence* in a poset $(P, \leq)$ is a subset $\{p_i | 0 \leq i, p_i \leq p_{i+1}\}$. A partially ordered set $(P, \leq)$ in which every ω-sequence has a least upper bound is called ω-*complete*, and if $(P, \leq)$ also has a least element, then $(P, \leq)$ is called a ω-*complete pointed partially ordered* set. An ω-complete pointed partially ordered set is called a *CPO* for short. A function $f : P \to Q$ between CPOs P and Q is *monotonic* whenever $p_i \leq p_j$ implies $f(p_i) \leq f(p_j)$ for all $p_i, p_j \in P$; and f is *continuous* whenever each sequence $\{p_i | 0 \leq i\} \subseteq P$ is a sequence $\{f(p_i) | 0 \leq i\} \subseteq Q$. Hence, continuous functions preserve LUBs and least elements. The category **CPO** has CPOs for objects and continuous functions for morphisms such that the set of functions $\{f : A \to B\}$ for each pair of objects A and B in **CPO** is a complete partially ordered set, and composition of functions preserves ordering: functions are ordered pointwise, i.e., $f_i \leq f_j$ whenever $f_i(a) \leq f_j(a)$ for all $a \in A$.

The importance of **CPO** is with regard to "fixed-points" and *structural induction*, which we do not get from **Set**. We just provide the intuition here (see [3,7] for formal details). A function $f : X \to X$ has a fixed point when $f(x) = x$ for some $x \in X$. Analogously, a functor $f : \mathbf{C} \to \mathbf{C}$ has a "fixed point" when $f(A) \cong A$. The presence of LUBs means that under certain general conditions recursive applications are guaranteed to converge. The presence of CPOs means that there is a continuous function from the natural numbers to each of its sequences starting from the least element. Hence, structural induction is based on induction over the natural numbers. The proofs that the various (un)folds are (anamorphisms) catamorphisms requires structural induction, and the presence of (final) initial F-(co)algebras depends of the functors F having fixed points. Our explanation for systematic recursive capacities in terms of these forms of universal constructions depends on **CPO**.

The partial order underlying our various applications of **CPO** is called *approximation ordering*, and is denoted $\sqsubseteq$ (see [7]). For sets (e.g., numbers, characters, etc.), approximation ordering is defined by:

$$x \sqsubseteq y \Leftrightarrow (x = \perp) \vee (x = y)$$

For pairs, it is defined by:

$$\bot \sqsubseteq (x, y) \qquad \text{and}$$
$$(x, y) \sqsubseteq (x', y') \Leftrightarrow (x \sqsubseteq x') \wedge (y \sqsubseteq y')$$

For lists, it is defined by:

$$\bot \sqsubseteq xs \qquad \text{and}$$
$$[\,] \sqsubseteq xs \Leftrightarrow xs = [\,]$$
$$(x \cdot xs) \sqsubseteq (y \cdot ys) \Leftrightarrow (x \sqsubseteq y) \wedge (xs \sqsubseteq ys)$$

The bottom element $\bot$ is interpreted as the "undefined", or "unknown" value. Since morphisms in **CPO** preserve points, partially defined functions are effectively total functions in this category. The element $\bot$ is also used for partially known information: e.g., a partially completed guest list is constructed as: $\mathit{John} \cdot \mathit{Mary} \cdot \mathit{Sue} \cdot \bot$. Moreover, by the definition of $\sqsubseteq$, we have, e.g., $\mathit{John} \cdot \mathit{Mary} \cdot \bot \sqsubseteq \mathit{John} \cdot \mathit{Mary} \cdot [\mathit{Sue}]$, since $\bot \sqsubseteq xs$ for all xs in the set of lists, which accords with our intuition that the second list provides more information about the coming guests than the first. Structural induction over lists, for example, proceeds by proving one or more base cases, typically involving $\bot$ and $[\,]$, assuming an arbitrary list xs, and then proving the induction step, $x \cdot xs$. So, the sequence $\bot \leq [\,] \leq \ldots xs \leq x \cdot xs$ corresponds to $0 \leq 1 \leq \ldots n \leq n + 1$, assuming an approximation ordering via list reversal: i.e., $sx \sqsubseteq_r sy \Leftrightarrow xs \sqsubseteq ys$, where sx (sy) is xs (ys) in reverse order. Structural induction over trees proceeds analogously, noting that tree order is related by pair order. Since $\bot$ is preserved we omit proofs involving this case.

Duals

Category theory constructs often come in pairs by reversing the direction of the relevant arrows.

Definition (Opposite category). Given a category $\mathbf{C}$, the *opposite category* $\mathbf{C}^{\mathbf{op}}$ is the category whose objects are all the objects of $\mathbf{C}$, and whose morphisms are obtained by reversing the directions of the arrows in $\mathbf{C}$.

Definition (Dual). Given a definition, proposition, or proof in a category $\mathbf{C}$, its dual is the corresponding definition, proposition, or proof in the category $\mathbf{C}^{\mathbf{op}}$.

The following examples are pairs of dual constructs: (initial object, terminal object), (coproduct, product), and (couniversal morphism, universal morphism). An isomorphism is self-dual. F-algebra, initial algebra and catamorphism also have duals called F-coalgebra, final coalgebra and anamorphism (respectively), which we detail later.

Definition (Initial object). An *initial object* in a category **C** is an object, denoted 0, such that for every object A in **C** there exists a unique morphism $u : 0 \to A$ in **C**.

Example (Initial object). In **Set** and **CPO**, the empty set $\varnothing$ is the initial object, and the unique morphism from $\varnothing$ to A is the empty function.

Definition (Terminal object). A *terminal object* in a category **C** is an object, denoted 1, such that for every object A in **C** there exists a unique morphism $u : A \to 1$ in **C**.

Example (Terminal object). In **Set**, any singleton set $\{x\}$ is a terminal object, and the unique morphism from A to $\{x\}$ is the function that maps every element $a \in A$ to x. In this case, there are many terminal objects. In **CPO**, the terminal object is the set $\{\bot\}$, for which $\bot$ is also the LUB.

Isomorphisms

Universal constructions are said to be *unique up to a unique isomorphism.* Here we explain what this expression means.

Definition (Isomorphism). A morphism $f : A \to B$ is an *isomorphism* if and only if there exists a morphism $g : B \to A$, such that $g \circ f = 1_A$ and $f \circ g = 1_B$. If g exists, then it is said to be the *inverse* of f (also denoted f^{-1}). If $f : A \to B$ is an isomorphism, then A is said to be *isomorphic* to B, written $A \cong B$.

In category theory, where the focus is on the relationships between objects (i.e., the morphisms), rather than the specifics of the objects themselves, one often only requires objects to be identified up to an isomorphism. That is, isomorphic objects are regarded as essentially the "same" object. For example, in a category **C** with terminal objects, all such objects are isomorphic (as proved below), and so are regarded as the same object, denoted by 1. In **Set**, where every singleton set is a terminal object, "the" terminal object is often denoted as $\{*\}$, where $*$ is an unnamed object, when we don't need its identity. Obviously, the isomorphism from singleton set $\{a\}$ to singleton set $\{b\}$ is the unique map $f : a \mapsto b$, which has the inverse $g : b \mapsto a$. In general, any two sets with the same number of objects are isomorphic. However, for $n > 1$, the isomorphism is not unique. For example, for sets $\{a_1, a_2\}$ and $\{b_1, b_2\}$, the

map $f_1 : a_1 \mapsto b_1, a_2 \mapsto b_2$ is an isomorphism, and so too is the map $f_2 : a_1 \mapsto b_2, a_2 \mapsto b_1$, though $f_1 \neq f_2$. Hence, we have the distinction between *unique up to an isomorphism*, and *unique up to a unique isomorphism.*

Proposition Terminal objects are unique up to a unique isomorphism.

Proof Suppose T and T' are two terminal objects. By definition (of terminal objects) there must exist unique morphisms $u : T' \to T$ and $v : T \to T'$. Consider the composition $v' \circ u : T \to T$. Since every object must have an identity map, this composition can only be the identity, i.e., $v' \circ u = 1_T$. Exchanging the roles of u and v yields $u \circ v = 1_{T'}$ by the same argument. Therefore u is an isomorphism. Since u is unique, it is a unique isomorphism, and likewise for v. □

Proposition Initial objects are unique up to a unique isomorphism.

Proof Duality!—replace the word terminal by initial, and turn all the arrows around, in the proof above, and you have a proof that initial objects are unique up to a unique isomorphism. □

(Co)Products

Definition (Product of objects). A *product of objects* A and B in category $\mathbf{C}$ is, up to unique isomorphism, an object P (also denoted $A \times B$) together with two morphisms (sometimes called *projections*) $p_1 : P \to A$ and $p_2 : P \to B$, jointly expressed as (P, p_1, p_2), such that for every object $Z \in |\mathbf{C}|$ and pair of morphisms $f : Z \to A$ and $g : Z \to B$ there exists a unique morphism $u : Z \to P$, also denoted $\langle f, g \rangle$, such that the following diagram commutes:

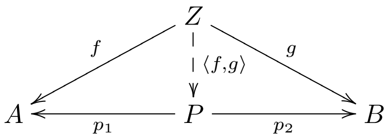

(1)

By a straightforward extension, the *finite product* of n objects $A_1, \ldots, A_n$ is $(A_1 \times \cdots \times A_n, p_1, \ldots, p_n)$.

Example (Product). The familiar Cartesian product of sets has this property in **Set**. In **CPO**, the product of two CPOs $(A, \leq)$ and $(B, \leq)$ is the Cartesian product $(A \times B)$ of their underlying sets, where $(\bot, \bot)$ is the bottom element, together with the partial order defined for pairs: i.e., $(a, b) \sqsubseteq (a', b') \Leftrightarrow (a \sqsubseteq a') \wedge (b \sqsubseteq b')$ for all $a, a' \in A$ and $b, b' \in B$. Not all categories have (all) products.

Definition (Coproduct of objects). A *coproduct of objects* A and B in category $\mathbf{C}$ is, up to unique

isomorphism, an object Q (also denoted $A+B$) together with two morphisms $q_1 : A \to Q$ and $q_2 : B \to Q$, jointly expressed as (Q, q_1, q_2), such that for every object $Z \in |\mathbf{C}|$ and pair of morphisms $f : A \to Z$ and $g : B \to Z$ there exists a unique morphism $u : Z \to P$, also denoted $[f, g]$, such that the following diagram commutes:

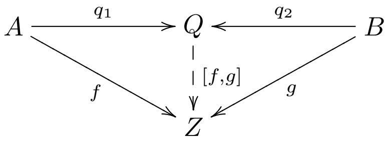

(2)

By a straightforward extension, the *finite coproduct* of n objects $A_1, \ldots, A_n$ is $(A_1 + \cdots + A_n, q_1, \ldots, q_n)$.

Example (Coproduct). In **Set**, the coproduct (up to a unique isomorphism) is *disjoint union.* The disjoint union of two sets A and B, denoted $A \uplus B$, is the set $\{(1, a)|a \in A\} \cup \{(2, b)|b \in B\}$. In **CPO**, the coproduct is the disjoint union, defined analogously as the disjoint union of the underlying sets (with their respective bottom elements removed), plus the bottom element, i.e., $\{\bot\} \cup (A^+ \uplus B^+)$, where A^+ and B^+ are sets A and B depleted of their bottom elements. The associated partial order is: $\bot \sqsubseteq (1, a)$, $\bot \sqsubseteq (2, b)$, and $a \sqsubseteq a' \Rightarrow (1, a) \sqsubseteq (1, a')$ and $b \sqsubseteq b' \Rightarrow (2, b) \sqsubseteq (2, b')$ for all $a, a' \in A^+$ and $b, b' \in B^+$. Not all categories have (all) coproducts.

Definition (Product of morphisms). A *product of morphisms* $f_1 : A_1 \to B_1$ and $f_2 : A_2 \to B_2$ is the unique morphism $f_1 \times f_2 : A_1 \times A_2 \to B_1 \times B_2$ such that the following diagram commutes:

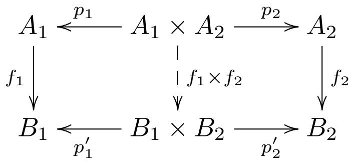

(3)

Such a unique morphism is guaranteed to exist by the definition of products (if the products exist). Finite products of n morphisms, denoted $f_1 \times \cdots \times f_n : A_1 \times \cdots \times A_n \to B_1 \times \cdots \times B_n$, are also defined by a straight-forward extension.

Definition (Coproduct of morphisms). A *coproduct of morphisms* $f_1 : A_1 \to B_1$ and $f_2 : A_2 \to B_2$

is the unique morphism $f_1 + f_2 : A_1 + A_2 \to B_1 + B_2$ such that the following diagram commutes:

$$\begin{array}{ccccc} A_1 & \xrightarrow{q_1} & A_1 + A_2 & \xleftarrow{q_2} & A_2 \\ {\scriptstyle f_1}\downarrow & & \downarrow{\scriptstyle f_1+f_2} & & \downarrow{\scriptstyle f_2} \\ B_1 & \xrightarrow{q'_2} & B_1 + B_2 & \xleftarrow[q'_2]{} & B_2 \end{array} \tag{4}$$

Such a unique morphism is guaranteed to exist by the definition of coproducts (if the coproducts exist). Finite coproducts of n morphisms, denoted $f_1 + \cdots + f_n : A_1 + \cdots + A_n \to B_1 + \cdots + B_n$, are also defined by a straight-forward extension.

Functors

Definition (Functor). A *functor* $F : \mathbf{C} \to \mathbf{D}$ is a map from a category $\mathbf{C}$ to a category $\mathbf{D}$ that associates each object A in $\mathbf{C}$ with an object $F(A)$ in $\mathbf{D}$; and each morphism $f : A \to B$ in $\mathbf{C}$ with a morphism $F(f) : F(A) \to F(B)$ in $\mathbf{D}$, and is structure-preserving in that $F(1_A) = 1_{F(A)}$ for each object A in $\mathbf{C}$; and $F(g \circ_{\mathbf{C}} f) = F(g) \circ_{\mathbf{D}} F(f)$ for all morphisms $f : A \to B$ and $g : B \to C$.

Example (Functor). A common example of a functor is a "forgetful" functor, such as the functor $U : \mathbf{Vec} \to \mathbf{Set}$, which maps a vector space to its underlying set, and a vector space morphism (that is, a linear mapping) to the underlying function. The following definitions give further examples of functors.

Definition (Isofunctor). A functor $F : \mathbf{C} \to \mathbf{D}$ is an *isofunctor* if and only if there exists a functor $G : \mathbf{D} \to \mathbf{C}$ such that $G \circ F = 1_{\mathbf{C}}$ and $F \circ G = 1_{\mathbf{D}}$, where $1_{\mathbf{C}}$ and $1_{\mathbf{D}}$ are identity functors.

Definition (Constant functor). A *constant functor* $K_D : \mathbf{C} \to \mathbf{D}; C \mapsto D, f \mapsto 1_D$ maps all objects C and morphisms f in $\mathbf{C}$ to the same object D and identity morphism 1_D in $\mathbf{D}$.

Definition (Identity functor). An *identity functor* $I : \mathbf{C} \to \mathbf{C}; C \mapsto C, f \mapsto f$ maps every object C and morphism f in $\mathbf{C}$ to itself.

A *(co)product of functors* is defined, because of its close relationship to *polynomial functors*, which form the basis of the F-algebras we use in our examples of recursive domains.

Definition (Product of functors—pointwise). A *pointwise (finite) product of functors* $F_i : \mathbf{C} \to \mathbf{D}$,

$i \in \{1, \dots, n\}$ is the functor $F_1 \times \cdots \times F_n$ indicated in the following diagram:

$$\mathbf{C} \xrightarrow{F_1 \times \cdots \times F_n} \mathbf{D} \tag{5}$$

$$\begin{array}{ccc} C_1 & & F_1(C_1) \times \cdots \times F_n(C_1) \\ \downarrow f & \longmapsto & \downarrow F_1(f) \times \cdots \times F_n(f) \\ C_2 & & F_1(C_2) \times \cdots \times F_n(C_2) \end{array}$$

where $F_i(C_i)$ has a set-like structure, and the product in $\mathbf{D}$ coincides with the Cartesian product of sets, which is the case for the categories that we use.

Definition (Coproduct of functors—pointwise). A *pointwise (finite) coproduct of functors* F_i : $\mathbf{C} \to \mathbf{D}$, $i \in \{1, \dots, n\}$ is the functor $F_1 + \cdots + F_n$ indicated in the following diagram:

$$\mathbf{C} \xrightarrow{F_1 + \cdots + F_n} \mathbf{D} \tag{6}$$

$$\begin{array}{ccc} C_1 & & F_1(C_1) + \cdots + F_n(C_1) \\ \downarrow f & \longmapsto & \downarrow F_1(f) + \cdots + F_n(f) \\ C_2 & & F_1(C_2) + \cdots + F_n(C_2) \end{array}$$

where $F_i(C_i)$ has a set-like structure, and the coproduct in $\mathbf{D}$ coincides with the disjoint union of sets, which is the case for the categories that we use.

Definition (Composition of functors). Given functors $F : \mathbf{C} \to \mathbf{D}$ and $G : \mathbf{D} \to \mathbf{E}$, the *composition of functors* $G \circ F : \mathbf{C} \to \mathbf{E}$ is given by $G \circ F : C \mapsto G(F(C)), f \mapsto G(F(f))$.

Definition (Cat). The category **Cat** has small categories for objects and functors for morphisms. (The technical distinction between small categories and otherwise need not concern us here, see [1].) Hence, composition is functor composition.

Definition (Endofunctor). An *endofunctor* $F : \mathbf{C} \to \mathbf{C}$ is a functor whose domain and codomain are the same category $\mathbf{C}$.

Definition (Polynomial functor). A *polynomial functor* is a constant functor, an identity functor, or a (co)product or composition of polynomial functors. Any polynomial functor is equivalent to one of the form $\sum_{i=0}^{n} K_{C_i} \times I^i \cong K_{C_0} + K_{C_1} \times I + K_{C_2} \times I^2 + \cdots + K_{C_n} \times I^n$, where $(+)\times$ is the (co)product of functors operator, and I^2 is equivalent to $I \times I$, etc. (Note that $K_{C_0} \times I^0 \cong K_{C_0}$.)

A product of categories is also defined, because it is needed for F-algebras.

Definition (Product of categories). Given categories $\mathbf{C}$ and $\mathbf{D}$, the *product of categories* $\mathbf{C} \times \mathbf{D}$ has pairs (C, D) as objects and pairs $(f, g) : (C_1, D_1) \to (C_2, D_2)$ as morphisms, where C and $f : C_1 \to C_2$ are in $\mathbf{C}$, and D and $g : D_1 \to D_2$ are in $\mathbf{D}$. Identity morphisms and composition are component-wise. It is routine to check that $\mathbf{C} \times \mathbf{D}$ is a category, and that $(\mathbf{C} \times \mathbf{D}, F_1, F_2)$ is a product in $\mathbf{Cat}$, where functors (projections) F_1 and F_2 return the first and second objects (morphisms) of each pair of objects (morphisms) in $\mathbf{C} \times \mathbf{D}$.

Definition (Bifunctor). A *bifunctor* $F(-, -) : \mathbf{C} \times \mathbf{D} \to \mathbf{E}; (C, D) \mapsto E, (f, g) \mapsto h$ is a functor with two arguments, mapping pairs of objects and pairs of morphisms from a product of categories $\mathbf{C} \times \mathbf{D}$ to objects and morphisms in a category $\mathbf{E}$. A *bifunctor fixed at first-argument object* A in $\mathbf{C}$ is a functor of the form $F_A = F(A, -) : \mathbf{D} \to \mathbf{E}; D \mapsto F(A, D), g \mapsto F(1_A, g)$, where D is an object and $g : D_1 \to D_2$ is a morphism in $\mathbf{D}$.

Definition (Parameterized functor). Given a bifunctor $F : \mathbf{C} \times \mathbf{D} \to \mathbf{E}$ and an object A in $\mathbf{C}$, a *parameterized functor* is the functor $F_A : \mathbf{D} \to \mathbf{E}$ of the form $F(A, -)$ defined above.

Example (Parameterized functor). An example of a functor $F : \mathbf{Set} \times \mathbf{Set} \to \mathbf{Set}$ parameterized by A is the polynomial functor $F_A = K_1 + K_A \times I : \mathbf{Set} \to \mathbf{Set}, S \mapsto 1 + A \times S, f \mapsto 1_1 + 1_A \times f$. This functor is used to recursively constructs sets of lists of type A, starting with the singleton set $\{[\,]\}$ containing the empty list $[\,]$. (Recall that in $\mathbf{Set}$, any singleton set is a terminal object, denoted 1.) So, for example, $F_A(1) = K_1(1) + K_A(1) \times I(1) = 1 + A \times 1 \cong 1 + A$ (essentially, corresponding to a set containing the empty list and all one-element lists). Since $1 + A$ is in the domain of F_A we also have $F_A(1 + A) = 1 + A \times (1 + A) \cong 1 + A + A \times A$ (essentially, corresponding to a set containing the empty list, all one-element lists, and all two-element lists). In this way, F_A constructs lists of arbitrary lengths. Functions over these lists are similarly constructed recursively. However, their relevance to recursive domains requires additional definitions, so examples are deferred to the next section.

Natural transformations

Definition (Natural transformation). A *natural transformation* $\eta : F \dot{\rightarrow} G$ between a domain functor $F : \mathbf{C} \rightarrow \mathbf{D}$ and a codomain functor $G : \mathbf{C} \rightarrow \mathbf{D}$ consists of $\mathbf{D}$−maps $\eta_A : F(A) \rightarrow G(A)$ for each object A in $\mathbf{C}$, such that $G(f) \circ \eta_A = \eta_B \circ F(f)$, as indicated by commutative diagram

$$\begin{array}{ccc} F(A) & \xrightarrow{\eta_A} & G(A) \\ {\scriptstyle F(f)}\downarrow & & \downarrow{\scriptstyle G(f)} \\ F(B) & \xrightarrow[\eta_B]{} & G(B) \end{array} \tag{7}$$

Definition (Natural isomorphism/natural equivalence). A natural transformation is a *natural isomorphism*, or *natural equivalence* if and only if each η_A is an isomorphism. That is, for each $\eta_A : F(A) \rightarrow G(A)$ there exists a $\eta_A^{-1} : G(A) \rightarrow F(A)$ such that $\eta_A^{-1} \circ \eta_A = 1_{F(A)}$ and $\eta_A \circ \eta_A^{-1} = 1_{G(A)}$.

Natural transformations are morphisms of functors, and so can be composed. Note that isomorphic functor and isofunctor are not the same thing. Isomorphic functors are functors that are isomorphic to each other and the map between them is a natural equivalence. Isofunctors are maps between isomorphic categories. We will provide examples of natural transformations when we investigate the relationship between functors that underlie different recursive domains.

F-algebras, initial algebras, and catamorphisms

Here we provide the definition of a category of F-algebras. Then, we build up category-theoretic models in terms of F-algebras for three recursive domains involving numbers, lists and trees. In each case, we start with a (bi)functor, possibly parameterized by an object identifying the type of the elements to which the corresponding operations apply, yielding a polynomial functor F that specializes to operations pertaining to the respective domains.

Definition (*F*-algebra). For an endofunctor $F : \mathbf{C} \rightarrow \mathbf{C}$, an *F-algebra* is a pair (A, α), where A is an object and $\alpha : F(A) \rightarrow A$ is a morphism in $\mathbf{C}$.

Example (*F*-algebra). Let $\mathbf{C}$ be the category $\mathbf{Set}$, $F : \mathbf{Set} \rightarrow \mathbf{Set}$ be the functor $F : S \mapsto S \times S, f \mapsto f \times f$, A be the set of natural numbers $\mathbb{N}$ (so, $\mathbb{N} \in |\mathbf{Set}|$), and $\alpha : \mathbb{N} \times \mathbb{N} \rightarrow \mathbb{N}$ be the morphism $\alpha : (m, n) \mapsto m + n$. Then, $(\mathbb{N}, \alpha)$ is an F-algebra.

Definition (*F*-algebra homomorphism). An *F-algebra homomorphism* $h : (A, \alpha) \to (B, \beta)$ is a morphism $h : A \to B$ (in $\mathbf{C}$) such that the following diagram commutes:

$$\begin{array}{ccc} F(A) & \xrightarrow{\alpha} & A \\ {\scriptstyle F(h)}\downarrow & & \downarrow{\scriptstyle h} \\ F(B) & \xrightarrow[\beta]{} & B \end{array} \tag{8}$$

Example (*F*-algebra homomorphism). Let (A, α) be the F-algebra $(\mathbb{N}, \alpha)$ from the previous example, (B, β) be the F-algebra $(\mathbb{N}, \beta)$, where $\beta : (n, m) \mapsto nm$ (i.e., multiplication), and $h : \mathbb{N} \to \mathbb{N}$ be the power function $h : n \mapsto 2^n$. Then, h is an F-algebra homomorphism (since $2^{n+m} = 2^n 2^m$).

F-algebra homomorphisms can be composed, and there are identity F-algebra homomorphisms. Hence, the collection of F-algebras and F-algebra homomorphisms is a category.

Definition (Category of *F*-algebras). For any endofunctor $F : \mathbf{C} \to \mathbf{C}$, the F-algebras form the category $\mathbf{Alg}(F)$ with F-algebras (A, α) for objects, and F-algebra homomorphisms $h : (A, \alpha) \to (B, \beta)$ for morphisms.

Definition (Initial algebra). An *initial F-algebra* (A, in), hereafter simply called an *initial algebra*, is an initial object in the category of F-algebras $\mathbf{Alg}(F)$. That is, there exists a unique F-algebra homomorphism from (A, in) to every F-algebra in $\mathbf{Alg}(F)$.

The presence of an initial algebra in a category $\mathbf{Alg}(F)$ depends on the particular functor F, and is not guaranteed to exist in every case.

Proposition. If $F : \mathbf{C} \to \mathbf{C}$ is a polynomial functor, then the category of F-algebras $\mathbf{Alg}(F)$ has an initial algebra.

Proof. See [3], chapters 10 and 11.

Definition (Catamorphism). A *catamorphism* $h : (A, \mathit{in}) \to (B, \beta)$ is the unique F-algebra homomorphism from initial F-algebra (A, in) to F-algebra (B, β). That is, $h \circ \mathit{in} = \beta \circ F(h)$ and the uniquely specified h for each such β is denoted cata β (i.e., $h = \text{cata } \beta$), as indicated in the following diagram:

$$\begin{array}{ccc} F(A) & \xrightarrow{\mathit{in}} & A \\ {\scriptstyle F(\text{cata } \beta)}\downarrow & & \downarrow{\scriptstyle \text{cata } \beta} \\ F(B) & \xrightarrow[\beta]{} & B \end{array} \tag{9}$$

where cata β is also denoted $(\!|\beta|\!)$ using *banana brackets* (see [4]).

The following result, due to Lambek [2], is important to our explanation of systematicity.

Lemma (Lambek). Given a functor $F : \mathbf{C} \to \mathbf{C}$ as the basis for a category of F-algebras $\mathbf{Alg}(F)$ with an initial algebra (A, in), then $in : F(A) \to A$ is a unique isomorphism $(F(A) \cong A)$.

Proof. We must show that there exists a morphism $h : A \to F(A)$ such that $in \circ h = 1_A$ (right inverse) and $h \circ in = 1_{F(A)}$ (left inverse), and that in is unique. Right inverse: Consider the diagram

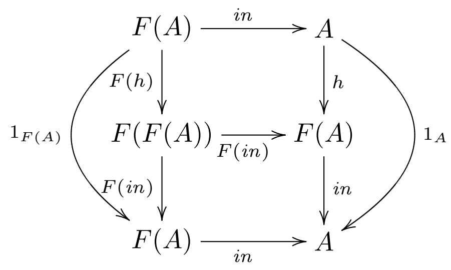

(10)

The existence of $h : A \to F(A)$ such that the upper inner square commutes is guaranteed, since $(F(A), F(in))$ is an F-algebra (by endofunctor F) and (A, in) is an initial algebra in $\mathbf{Alg}(F)$. The arrows in the lower inner square are already given, since F is a functor. Hence the outer square commutes too. Thus, $in \circ h : A \to A$ is an F-algebra homomorphism. 1_A and $1_{F(A)}$ also make the outer square commute (see curved arrows). Since (A, in) is an initial algebra, the F-algebra homomorphism from A to A is unique, so $in \circ h = 1_A$.

Left inverse: From the upper inner commutative square of Diagram 10, we have $h \circ in = F(in) \circ F(h)$ (since F is a functor) $= F(in \circ h) = F(1_A) = 1_{F(A)}$. That is, $h \circ in = 1_{F(A)}$. So, in is an isomorphism. Since h is unique (being a catamorphism) and $in = h^{-1}$, in must also be unique. That is, in is the unique (F-algebra) isomorphism $F(A) \to A$. □

Examples of initial algebras and associated catamorphisms are given in the subsequent sections. An initial algebra (A, in) in $\mathbf{Alg}(F)$ on a functor $F : \mathbf{C} \to \mathbf{C}$ is an example of a universal construction. Specifically, it is an instance of a couniversal morphism (see Diagrams 43). That is, given an object $X = F(A)$ in $\mathbf{C}$ and a (identity) functor $F = I : \mathbf{C} \to \mathbf{C}$, (A, in) is a couniversal morphism from $F(A)$ to I, since for every object $Y = B$ in $\mathbf{C}$ and every morphism $f : X \to F(Y)$, which is the morphism $\beta \circ F(h) : F(A) \to \beta$ (in Diagram 9), there exists a unique morphism, by definition of catamorphism,

$h = (\!|\beta|\!) : A \to B$ (see Diagram 9) such that the corresponding diagram

$$\mathbf{A} \xrightarrow{T_{F(A)}} \mathbf{C} \qquad \mathbf{C} \xleftarrow{I} \mathbf{C} \qquad (F(A) \downarrow I) \tag{11}$$

$$\begin{array}{ccccc} F(A) \xrightarrow{in} & A & A & & (A, in) \\ & \Big\downarrow (\!|\beta|\!) & \Big\downarrow (\!|\beta|\!) & & (\!|\beta|\!) \Big\downarrow \\ {\scriptstyle \beta \circ F((\!|\beta|\!))} \searrow & B & B & & (B, \beta \circ F((\!|\beta|\!))) \end{array}$$

commutes.

Numbers

For computation with regard to numbers, we start with the polynomial functor $F = K_1 + I : \mathbf{CPO} \to \mathbf{CPO}; S \mapsto 1 + S, f \mapsto 1_1 + f$, where $K_1 : S \mapsto 1, f \mapsto 1_1$ is a constant functor respectively sending all objects S and morphisms f to terminal object 1 and its identity morphisms 1_1, and I is the identity functor for $\mathbf{CPO}$. In this case, the functor is not parameterized. The F-algebras for the category $\mathbf{Alg}(F)$ are the pairs $(S, [\mathsf{I}_v, f])$, where S is a set, $[\mathsf{I}_v, f] : 1 + S \to S$ is a function from coproduct (disjoint union) $1 + S$ to S, $\mathsf{I}_v : 1 \to S$ is a nullary function (equivalently, a constant) returning $v \in S$, and $f : s \mapsto s'$ is a unary function. An initial algebra in this category is $(N, [\mathit{zero}, \mathit{succ}])$, where N is a set that corresponds to the natural numbers ($\mathbb{N}$), $[\mathit{zero}, \mathit{succ}] : 1 + N \to N$, $\mathit{zero} : 1 \to N$ is a nullary function (equivalently, a constant) returning the element $\mathit{Zero} \in N$, and $\mathit{succ} : N \to N; n \mapsto \mathit{succ}(n)$ is a unary function returning the successor of element $n \in N$. Initial algebra $(N, [\mathit{zero}, \mathit{succ}])$ is an F-algebra $(S, [\mathsf{I}_v, f])$, where S is the set N, I_v is the nullary function $\mathit{zero} = \mathsf{I}_{\mathit{Zero}}$, and f is the unary function succ. Thus, N contains Zero, $\mathit{succ}(\mathit{Zero})$, $\mathit{succ}(\mathit{succ}(\mathit{Zero}))$, etc; and $\mathit{succ}(\mathit{Zero})$ corresponds to 1, $\mathit{succ}(\mathit{succ}(\mathit{Zero}))$ to 2, and so on. This characterization of the natural numbers based on *Zero* and *succ* relies on the well-known work of Peano.

There is a catamorphism from initial algebra $(N, [\mathit{zero}, \mathit{succ}])$ to each F-algebra $(S, [\mathsf{I}_v, f])$ in $\mathbf{Alg}(F)$ called *foldN* (i.e., fold for N), defined as $\mathit{foldN}\,[\mathsf{I}_v, f] : N \to S, \mathit{Zero} \mapsto v, \mathit{succ}(n) \mapsto \mathit{foldN}\,[\mathsf{I}_{f(v)}, f]\,(n)$, where $f : S \to S$ is a unary function, and $v \in S$ is a constant. We also define $\mathit{foldN}'\,[\mathsf{I}_v, f] : N \to S, \mathit{Zero} \mapsto v, \mathit{succ}(n) \mapsto f(\mathit{foldN}'\,[\mathsf{I}_v, f]\,(n))$.

Proposition. $\mathit{foldN}\,[\mathsf{I}_v, f] = \mathit{foldN}'\,[\mathsf{I}_v, f]$.

Proof (by induction). Proof by induction has a common pattern consisting of a proof for the base case (Base), an inductive assumption for an arbitrary non-base case (Assumption), and a proof of the case that succeeds the assumption case (Induction step). Since we are working in the category **CPO**, full induction also requires proving the cases that involve the bottom element $\perp$ (see [7]). The proofs provided here are easily extended to such cases, so the details are omitted.

Base—*Zero*: Immediate.

Assumption—n: $\mathit{foldN}'\,[\mathsf{l}_v, f]\,(n) = \mathit{foldN}\,[\mathsf{l}_v, f]\,(n)$

Induction step—$\mathit{succ}(n)$:

$$\begin{aligned}
\mathit{foldN}[\mathsf{l}_v, f]\,(\mathit{succ}(n)) &= \mathit{foldN}[\mathsf{l}_v, f] \circ [\mathit{zero}, \mathit{succ}](n) && \text{(composition)}\\
&= f(\mathit{foldN}[\mathsf{l}_v, f](n)) && \text{(F-algebra morphism, see Diagram 12)}\\
&= f(\mathit{foldN}'[\mathsf{l}_v, f](n)) && \text{(by the inductive assumption)}\\
&= \mathit{foldN}'[\mathsf{l}_v, f](\mathit{succ}(n)) && \text{(definition of } \mathit{foldN}'\text{)} \qquad \square
\end{aligned}$$

The condition for $\mathit{foldN}\,[\mathsf{l}_v, f]$ to be an F-algebra morphism is indicated by the commutative diagram

$$\begin{array}{ccc}
1 + N & \xrightarrow{[\mathit{zero},\, \mathit{succ}]} & N \\
{\scriptstyle 1_1 + \mathit{foldN}\,[\mathsf{l}_v, f]}\downarrow & & \downarrow{\scriptstyle \mathit{foldN}\,[\mathsf{l}_v, f]} \\
1 + S & \xrightarrow[{[\mathsf{l}_v, f]}]{} & S
\end{array} \qquad (12)$$

Next, we show that $\mathit{foldN}'[\mathsf{l}_v, f]$ (therefore, $\mathit{foldN}[\mathsf{l}_v, f]$) is a catamorphism. We prove for $\mathit{foldN}'[\mathsf{l}_v, f]$, because the proof is slightly simpler than for $\mathit{foldN}[\mathsf{l}_v, f]$.

Proposition. $\mathit{foldN}'[\mathsf{l}_v, f]$ (and $\mathit{foldN}[\mathsf{l}_v, f]$) is a catamorphism.

Proof (by induction). First, we need to show that $\mathit{foldN}'\,[\mathsf{l}_v, f]$ exists (i.e., Diagram 12 commutes). Then, we need to show that $\mathit{foldN}'\,[\mathsf{l}_v, f]$ is unique (i.e., if $g : N \to S$ is other F-algebra homomorphism, then $g = \mathit{foldN}'\,[\mathsf{l}_v, f]$).

Existence (commutativity):

Base—$(1,*)$: i.e., where $(1,*) \in 1+N$ is being mapped, as opposed to $(2,n)$ where $n \in N$.

$$\begin{aligned}
\curvearrowright\!: \mathit{foldN}'[\mathsf{l}_v, f] \circ [\mathit{zero}, \mathit{succ}](1,*) &= \mathit{foldN}'[\mathsf{l}_v, f](\mathit{Zero}) && \text{(definition of } [\mathit{zero}, \mathit{succ}]) \\
&= v && \text{(definition of } \mathit{foldN}') \\
\curvearrowleft\!: [\mathsf{l}_v, f] \circ (1_1 + \mathit{foldN}'[\mathsf{l}_v, f])(1,*) &= [\mathsf{l}_v, f](1,*) && \text{(definition of } 1_1 + \mathit{foldN}'[\mathsf{l}_v, f]) \\
&= v && \text{(definition of } [\mathsf{l}_v, f])
\end{aligned}$$

Assumption—$(2,n)$:

$$\begin{aligned}
\mathit{foldN}'[\mathsf{l}_v, f] \circ [\mathit{zero}, \mathit{succ}](2,n) &= [\mathsf{l}_v, f] \circ (1_1 + \mathit{foldN}'[\mathsf{l}_v, f])(2,n) && \text{(Diagram 12)} \\
\mathit{foldN}'[\mathsf{l}_v, f](\mathit{succ}(n)) &= f(\mathit{foldN}'[\mathsf{l}_v, f](n)) && \text{(LHS/RHS reductions, by def.)}
\end{aligned}$$

Induction step—$(2,(\mathit{succ}(n)))$:

$$\begin{aligned}
\curvearrowright\!: \mathit{foldN}'[\mathsf{l}_v, f] \circ [\mathit{zero}, \mathit{succ}](2,(\mathit{succ}(n))) &= \mathit{foldN}'[\mathsf{l}_v, f](\mathit{succ}(\mathit{succ}(n))) && \text{(definition of } [\mathit{zero}, \mathit{succ}]) \\
&= f(\mathit{foldN}'[\mathsf{l}_v, f](\mathit{succ}(n))) && \text{(definition of } \mathit{foldN}') \\
&= f(f(\mathit{foldN}'[\mathsf{l}_v, f](n))) && \text{(by inductive assumption)} \\
\curvearrowleft\!: [\mathsf{l}_v, f] \circ (1_1 + \mathit{foldN}'[\mathsf{l}_v, f])(2, \mathit{succ}(n)) &= [\mathsf{l}_v, f](\mathit{foldN}'[\mathsf{l}_v, f](\mathit{succ}(n))) && \text{(definition of } 1_1 + \dots) \\
&= [\mathsf{l}_v, f](f(\mathit{foldN}'[\mathsf{l}_v, f](n))) && \text{(by inductive assumption)} \\
&= f(f(\mathit{foldN}'[\mathsf{l}_v, f](n))) && \text{(definition of } [\mathsf{l}_v, f])
\end{aligned}$$

Uniqueness: Suppose g is an F-algebra homomorphism $g : N \to S$. The definition of F-algebra homomorphism forces $g(\mathit{Zero}) = v$, and $g(\mathit{succ}(n)) = f(g(n))$.

Base—*Zero*: $g(\mathit{Zero}) = v$, so $g(\mathit{Zero}) = \mathit{foldN}'[\mathsf{l}_v, f](\mathit{Zero})$.

Assumption—n: $g(n) = \mathit{foldN}\,[\mathsf{l}_v, f]\,(n)$.

Induction step—$succ(n)$:

$$\begin{aligned} g(succ(n)) &= f(g(n)) && \text{(definition of } g\text{)} \\ &= f(foldN'[\mathsf{I}_v, f]\,(n)) && \text{(by the inductive assumption)} \\ &= foldN'[\mathsf{I}_v, f]\,(succ(n)) && \text{(definition of } foldN'\text{)} \end{aligned}$$

Hence, $foldN[\mathsf{I}_v, f]$ is also a catamorphism. □

The two versions differ in their order of evaluation: *foldN* evaluates its argument before the recursive call, effectively progressing from *Zero* to argument n ; $foldN'$ evaluates its argument after the recursive call, effectively progressing from n to *Zero*.

We also define two versions of *foldn* (i.e., fold for numbers) to work directly with natural numbers. The definitions and proofs (not shown) follow the same patterns as for the two versions of *foldN*. They are defined as $foldn\,[\mathsf{I}_v, f] : \mathbb{N} \to S; 0 \mapsto v, (n+1) \mapsto foldn\,[\mathsf{I}_{f(v)}, f]\,(n)$, and $foldn'\,[\mathsf{I}_v, f] : \mathbb{N} \to S; 0 \mapsto v, (n+1) \mapsto f(foldn\,[\mathsf{I}_v, f]\,(n))$.

Examples

The initial algebra $(N, [zero, succ])$ is a model for the natural numbers. For example, the elements $Zero, succ(Zero), succ(succ(Zero)) \in N$ correspond to the elements $0, 1, 2 \in \mathbb{N}$, respectively. And, being an initial algebra, the catamorphisms from this algebra provide models for operations over natural numbers. For example, addition of two natural numbers is modeled as $plusN : N \times N \to N$, where $plusN\ m : n \mapsto foldN\,[\mathsf{I}_m, succ]\,(n)$. In effect, $plusN(m)(-)$ is a function that adds m to its $(-)$ argument. For example, $plusN\,(succ(Zero))(succ(Zero)) = succ(succ(Zero))$, which corresponds to $1 + 1 = 2$.

Lists

A list (e.g., constructed from the letters: ‘s’, ‘n’, ‘o’, and ‘c’) can be represented internally by a recursive construction that prepends items to the front of a list (as in “cons”), or by a recursive construction that postpends items to the back of a list (as in “snoc”). (By *postpend* we mean adding an *item* to the end of a list to create a new list, in contrast to *append*, which usually means adding a *list* to a list to create a new list in a functional programming language.) Each construction is an initial algebra in a particular category of F-algebras based on a specific functor. We provide both functors. Though

our focus is the more commonly used prepend construction, the results extend naturally (via a natural isomorphism) to the other version. The presence of multiple constructions raises the question, which we have alluded to in the main text, *How does the cognitive system know which algebra to use?* The process for constructing/destructing lists is intrinsically linked by a dual relationship between F-algebra and F-coalgebra. This relationship is detailed in the final section.

Prepend version: We start with a polynomial functor $F_A : \mathbf{CPO} \to \mathbf{CPO}$ parameterized by an object A in $\mathbf{CPO}$ as the basis for a category of algebras $\mathbf{Alg}(F_A)$ on functor F_A. An initial algebra in this category is a model for lists of objects of type A, and the catamorphisms from this initial algebra are models for recursive computations over such lists. The functor $F_A = K_1 + K_A \times I : \mathbf{CPO} \to \mathbf{CPO}; S \mapsto 1 + A \times S, f \mapsto 1_1 + 1_A \times f$, where $K_A : S \mapsto A, f \mapsto 1_A$ is a constant functor respectively sending all objects S to object A and all morphisms f to identity morphism 1_A. The F-algebras in the category $\mathbf{Alg}(F_A)$ on F_A are the objects $(S, [\mathsf{l}_v, f])$, where $[\mathsf{l}_v, f] : 1 + A \times S \to S$, $\mathsf{l}_v : 1 \to S$ always returns $v \in S$, and $f : A \times S \to S, (a, s) \mapsto s'$ is an arbitrary binary function. An initial algebra in this category is $(L, [\mathit{empty}, \mathit{cons}])$, where $[\mathit{empty}, \mathit{cons}] : 1 + A \times L \to L$, $\mathit{empty} : 1 \to L$ returns the empty list $[\,]$, and $\mathit{cons} : A \times L \to L; (a, l) \mapsto a \cdot l$ returns the list with element $a \in A$ prepended $(\cdot)$ to list $l \in L$. Initial algebra $(L, [\mathit{empty}, \mathit{cons}])$ is an F-algebra $(S, [\mathsf{l}_v, f])$, where S is the set of lists L (of type A), l_v is the function $\mathit{empty} = \mathsf{l}_{[\,]}$, and f is the binary function *cons*. (Historically, *cons* stands for "construct".)

Postpend version: This version is based on the polynomial functor $F_A = K_1 + I \times K_A : \mathbf{CPO} \to \mathbf{CPO}; S \mapsto 1 + S \times A, f \mapsto 1_1 + f \times 1_A$. The F-algebras in the category based on this functor are the objects $(S, [\mathsf{l}_v, f])$, where $[\mathsf{l}_v, f] : 1 + S \times A \to S$ and $f : S \times A \to S, (s, a) \mapsto s'$ is an arbitrary binary function. An initial algebra in this category is $(L, [\mathit{empty}, \mathit{snoc}])$, where $[\mathit{empty}, \mathit{snoc}] : 1 + L \times A \to L$, and $\mathit{snoc} : L \times A \to L; (l, a) \mapsto l \cdot a$ returns the list with element $a \in A$ postpended $(\cdot)$ to list $l \in L$. For the remainder of this section, we focus on the prepend version of lists, which is more commonly used in functional programming. Nonetheless, the postpend version may be more natural for particular cognitive capacities, which we also discuss in this section. The formal relationship between the two versions is given in the next section, where we also present the relationships between other F-algebras.

Somewhat like $\mathit{foldN}/\mathit{foldN}'$ ($\mathit{foldn}/\mathit{foldn}'$) for numbers, there are two recursively defined functions for processing prepend-constructed lists. For readers familiar with the functional programming language Haskell [8], they are called *foldl* (i.e., fold lists from the right/front) and *foldr* (i.e., fold lists from the left/back). Unlike the catamorphisms for number, though, only *foldr* is a general catamorphism

for prepend-constructed lists. The function *foldl* is equivalent to *foldr* only under certain restricted conditions (see below). If we require a catamorphism that processes lists from the left, then there is such a catamorphism for postpend-constructed lists, which we will call *foldL*. Since our primary concern is initial algebras and their associated catamorphisms, we will refer to *foldr* as foldL', by analogy with the naming convection $\mathit{foldN}/\mathit{foldN}'$ ($\mathit{foldn}/\mathit{foldn}'$) for numbers. Our interest in *foldl* is only expository. First, we present the definitions for *foldl* and *foldr* (foldL'). Then, we show that foldL' is a catamorphism, and finally, we present *foldL*, which is the general catamorphism for postpend-constructed lists.

The function *foldl* is defined as $\mathit{foldl}[\mathsf{l}_v, f] : L \to S; [\,] \mapsto v, (a \cdot l) \mapsto \mathit{foldl}\,[\mathsf{l}_{f(v,a)}, f]\,(l)$, where $f : S \times A \to S$ is a binary function, and $v \in S$ is a constant. The function *foldr* is defined as $\mathit{foldr}[\mathsf{l}_v, f] : [\,] \mapsto v, (a \cdot l) \mapsto f(a, \mathit{foldr}\,[\mathsf{l}_v, f]\,(l))$, where $f : A \times S \to S$ is a binary function, and $v \in S$ is a constant. If f is restricted to be an associative function, i.e., $S = A$, so $f : A \times A \to A$, and $f(a, f(b, c)) = f(f(a, b), c)$, then $\mathit{foldl}[\mathsf{l}_v, f] = \mathit{foldr}[\mathsf{l}_v, f]$ (see [7]).

Proposition. If $f : A \times S \to S$ is an arbitrary function, then $\mathit{foldL}'[\mathsf{l}_v, f]$ (i.e., $\mathit{foldr}[\mathsf{l}_v, f]$) is a catamorphism, indicated by the commutative diagram

$$\begin{array}{ccc} 1 + A \times L & \xrightarrow{[\mathit{empty},\, \mathit{cons}]} & L \\ {\scriptstyle 1_1 + 1_A \times \mathit{foldL}'[\mathsf{l}_v, f]} \downarrow & & \downarrow {\scriptstyle \mathit{foldL}'[\mathsf{l}_v, f]} \\ 1 + A \times S & \xrightarrow[{[\mathsf{l}_v, f]}]{} & S \end{array} \tag{13}$$

Proof (by induction). The proof follows the same pattern as the proof that $\mathit{foldN}/\mathit{foldN}'$ is a catamorphism: i.e., we first show that foldL' exists (Diagram 13 commutes), and then show uniqueness.

Existence (commutativity):

Base—$(1, *)$:

$$\begin{aligned} \curvearrowright\!: \mathit{foldL}'[\mathsf{l}_v, f] \circ [\mathit{empty}, \mathit{cons}](1, *) &= \mathit{foldL}'[\mathsf{l}_v, f]([\,]) && \text{(definition of } [\mathit{empty}, \mathit{cons}]) \\ &= v && \text{(definition of } \mathit{foldL}') \\ \curvearrowleft\!: [\mathsf{l}_v, f] \circ (1_1 + 1_A \times \mathit{foldL}'[\mathsf{l}_v, f])(1, *) &= [\mathsf{l}_v, f](1, *) && \text{(definition of } 1_1 + \mathit{foldL}'[\mathsf{l}_v, f]) \\ &= v && \text{(definition of } [\mathsf{l}_v, f]) \end{aligned}$$

Assumption—$(2,(a,l))$:

$$\mathit{foldL}'[\mathsf{I}_v,f]\circ[\mathit{empty},\mathit{cons}](2,(a,l)) = [\mathsf{I}_v,f]\circ(1_1+1_A\times\mathit{foldL}'[\mathsf{I}_v,f])(2,(a,l)) \qquad \text{(Diagram 13)}$$
$$\mathit{foldL}'[\mathsf{I}_v,f](a\cdot l) = f(a,\mathit{foldL}'[\mathsf{I}_v,f](l)) \qquad \text{(L/RHS reductions)}$$

Induction step—$(2,(a',(a\cdot l)))$: abbreviated to $(-)$

$$\begin{aligned}
\curvearrowright\!: \mathit{foldL}'[\mathsf{I}_v,f]\circ[\mathit{empty},\mathit{cons}](-) &= \mathit{foldL}'[\mathsf{I}_v,f](a'\cdot(a\cdot l)) && \text{(def. of } [\mathit{empty},\mathit{cons}]\text{)}\\
&= f(a',\mathit{foldL}'[\mathsf{I}_v,f](a\cdot l)) && \text{(definition of } \mathit{foldL}'\text{)}\\
&= f(a',f(a,\mathit{foldL}'[\mathsf{I}_v,f](l))) && \text{(inductive assumption)}\\
\curvearrowleft\!: [\mathsf{I}_v,f]\circ(1_1+1_A\times\mathit{foldL}'[\mathsf{I}_v,f])(-) &= [\mathsf{I}_v,f](2,(a',\mathit{foldL}'[\mathsf{I}_v,f](a\cdot l))) && \text{(definition of } 1_1+\dots\text{)}\\
&= [\mathsf{I}_v,f](2,(a',f(a,\mathit{foldL}'[\mathsf{I}_v,f](l)))) && \text{(inductive assumption)}\\
&= f(a',f(a,\mathit{foldL}'[\mathsf{I}_v,f](l))) && \text{(definition of } [\mathsf{I}_v,f]\text{)}
\end{aligned}$$

Uniqueness: Suppose we have an F-algebra homomorphism $g:L\to S$, indicated by the commutative diagram

$$\begin{array}{ccc}
1+A\times L & \xrightarrow{[\mathit{empty},\,\mathit{cons}]} & L\\
{\scriptstyle 1_1+1_A\times g}\downarrow & & \downarrow{\scriptstyle g}\\
1+A\times S & \xrightarrow[{[\mathsf{I}_v,f]}]{} & S
\end{array} \qquad (14)$$

Base—$[\,]$: $\curvearrowright$: $g\circ[\mathit{empty},\mathit{cons}](1,*) = g([\,])$. $\curvearrowleft$: $[\mathsf{I}_v,f]\circ(1_1+1_A\times g)(1,*) = [\mathsf{I}_v,f](1,*) = v$. Since g is an F-algebra homomorphism, $g([\,]) = v$.

Assumption—l: $g(l) = \mathit{foldL}'[\mathsf{I}_v,f](l)$.

Induction step—$(a\cdot l)$:

$$\begin{aligned}
g(a\cdot l) &= f(a,g(l)) && \text{(g is an F-algebra homomorphism, Diagram 14)}\\
&= f(a,\mathit{foldL}'[\mathsf{I}_v,f](l)) && \text{(by the inductive assumption)}\\
&= \mathit{foldL}'[\mathsf{I}_v,f](a\cdot l) && \text{(definition of } \mathit{foldL}'\text{)}
\end{aligned}$$

Therefore, $g = foldL' \, [\mathsf{I}_v, f]$. □

For functor $F_A : \mathbf{CPO} \to \mathbf{CPO}; S \mapsto 1 + S \times A, f \mapsto 1_1 + f \times 1_A$, the F-algebra homomorphism *foldL* is defined as $foldL[\mathsf{I}_v, f] : [\,] \mapsto v, (l \cdot a) \mapsto f(foldL[\mathsf{I}_v, f](l), a)$, where $f : S \times A \to S$ is a binary function, and $v \in S$ is a constant.

Proposition. If $f : S \times A \to S$ is an arbitrary function, then $foldL[\mathsf{I}_v, f]$ is a catamorphism, indicated by the commutative diagram

$$\begin{array}{ccc} 1 + L \times A & \xrightarrow{[empty,\, snoc]} & L \\ {\scriptstyle 1_1 + foldL[\mathsf{I}_v, f] \times 1_A} \big\downarrow & & \big\downarrow {\scriptstyle foldL[\mathsf{I}_v, f]} \\ 1 + S \times A & \xrightarrow[{[\mathsf{I}_v, f]}]{} & S \end{array} \tag{15}$$

Proof. Reverse the roles of the objects/morphisms associated with the products in the above proof. □

Examples

The following are concrete examples of F-algebras and associated catamorphisms. For the first and second examples, the parameter A to the functor F_A is the set of natural numbers $\mathbb{N}$, so the example deals with lists of natural numbers. For the third example, the parameter A is an arbitrary set. For the first example, we also include the postpend version, which extends analogously to the second and third examples. Note that $\mathbb{N}$ is also a CPO, with 0 as the bottom element, ∞ as the LUB and $\leq$ for numbers as the partial (total) order.

For the first example, we specialize object A in Diagram 13 to $\mathbb{N}$ to work with lists of natural numbers. If needed, we could also work with the set N as a model of the natural numbers (see Diagram 12), in which case the following examples can be straightforwardly translated. For expository purposes, $\mathbb{N}$ is more succinct. The functor $F_{\mathbb{N}} = K_1 + K_{\mathbb{N}} \times I : \mathbf{CPO} \to \mathbf{CPO}; S \mapsto 1 + \mathbb{N} \times S, f \mapsto 1_1 + 1_{\mathbb{N}} \times f$. An initial algebra in the category $\mathbf{Alg}(F_{\mathbb{N}})$ is the pair $(L, [empty, cons])$, where L is the set of lists of numbers, and *cons* is the corresponding function for constructing such lists. The pair $(\mathbb{N}, [\mathsf{I}_0, (+)])$, where $\mathsf{I}_0 : * \mapsto 0$ and $(+) : (x, y) \mapsto x + y$ is also an F-algebra in this category. The two algebras are related by

the catamorphism $\mathit{foldL}'[\mathsf{I}_0, (+)] : L \to \mathbb{N}$, and catamorphism is indicated in commutative diagram

$$\begin{array}{ccc} 1 + \mathbb{N} \times L & \xrightarrow{[\mathit{empty}, \mathit{cons}]} & L \\ {\scriptstyle 1_1 + 1_{\mathbb{N}} \times \mathit{foldL}'[\mathsf{I}_0,(+)]} \downarrow & & \downarrow {\scriptstyle \mathit{foldL}'[\mathsf{I}_0,(+)]} \\ 1 + \mathbb{N} \times \mathbb{N} & \xrightarrow[{[\mathsf{I}_0,(+)]}]{} & \mathbb{N} \end{array} \tag{16}$$

This catamorphism computes the sum of a list of numbers: 1, 2, 3, and 4, which is constructed as: $\mathit{cons}(4, \mathit{cons}(3, \mathit{cons}(2, \mathit{cons}(1, \mathit{empty}())))) = [4, 3, 2, 1]$. From the definition of foldL' we have, for list instance, $\mathit{foldL}'[\mathsf{I}_0, (+)]\,([[4, 3, 2, 1]) = 4 + (3 + (2 + (1 + (0))))) = 10$.

Prepend-constructed lists of natural numbers are based on the functor $F_{\mathbb{N}} : \mathbf{CPO} \to \mathbf{CPO}; S \mapsto 1 + S \times \mathbb{N}, f \mapsto 1_1 + f \times 1_{\mathbb{N}}$. An initial algebra in the category based on this functor is the pair $(L, [\mathit{empty}, \mathit{snoc}])$. The catamorphism for summing such lists is $\mathit{foldL}[\mathsf{I}_0, (+)] : L \to \mathbb{N}$, indicated in commutative diagram

$$\begin{array}{ccc} 1 + L \times \mathbb{N} & \xrightarrow{[\mathit{empty}, \mathit{snoc}]} & L \\ {\scriptstyle 1_1 + \mathit{foldL}[\mathsf{I}_0,(+)] \times 1_{\mathbb{N}}} \downarrow & & \downarrow {\scriptstyle \mathit{foldL}[\mathsf{I}_0,(+)]} \\ 1 + \mathbb{N} \times \mathbb{N} & \xrightarrow[{[\mathsf{I}_0,(+)]}]{} & \mathbb{N} \end{array} \tag{17}$$

The list: 1, 2, 3, and 4 is constructed as: $\mathit{snoc}(\mathit{snoc}(\mathit{snoc}(\mathit{snoc}(\mathit{empty}(), 1), 2), 3), 4) = [1, 2, 3, 4]$. The sum of this list is computed as: $\mathit{foldL}[\mathsf{I}_0, (+)]([1, 2, 3, 4]) = (((((0) + 1) + 2) + 3) + 4) = 10$.

For the second example, another F-algebra in the category $\mathbf{Alg}(F_{\mathbb{N}})$ is the pair $(\mathbb{N}, [\mathsf{I}_0, (\times)])$, where $\mathsf{I}_1 : * \mapsto 1$ and $(\times) : (x, y) \mapsto x \times y$, and catamorphism is indicated in commutative diagram

$$\begin{array}{ccc} 1 + \mathbb{N} \times L & \xrightarrow{[\mathit{empty}, \, \mathit{cons}]} & L \\ {\scriptstyle 1_1 + 1_{\mathbb{N}} \times \mathit{foldL}'[\mathsf{I}_1,(\times)]} \downarrow & & \downarrow {\scriptstyle \mathit{foldL}'[\mathsf{I}_1,(\times)]} \\ 1 + \mathbb{N} \times \mathbb{N} & \xrightarrow[{[\mathsf{I}_1,(\times)]}]{} & \mathbb{N} \end{array} \tag{18}$$

This catamorphism computes the product of a list of numbers. For instance, $\mathit{foldL}'[\mathsf{I}_1, (\times)]([4, 3, 2, 1]) = 4 \times (3 \times (2 \times (1 \times (1))))) = 24$.

For the third example, an F-algebra for an arbitrary set A is the pair $[\mathsf{I}_0, (\mathit{succ} \circ p_2)]$, where $\mathit{succ} : n \mapsto n + 1$ increments a natural number, and $p_2 : A \times \mathbb{N} \to \mathbb{N}$ is the projection onto the second factor. A

catamorphism for computing list length is indicated in commutative diagram

$$\begin{array}{ccc} 1 + A \times L & \xrightarrow{[empty,\, cons]} & L \\ {\scriptstyle 1_1 + 1_A \times foldL'[\mathsf{I}_0, (succ \circ p_2)]} \downarrow & & \downarrow {\scriptstyle foldL'[\mathsf{I}_0, (succ \circ p_2)]} \\ 1 + A \times \mathbb{N} & \xrightarrow[{[\mathsf{I}_0, (succ \circ p_2)]}]{} & \mathbb{N} \end{array} \tag{19}$$

For instance, $foldL'[\mathsf{I}_0, (succ \circ p_2)]\,([cat, dog, mouse]) = succ(succ(succ(0))) = 3$.

Some other examples of catamorphisms for the same category of F-algebras on functor F_A include:

smallest The smallest number of a list: $foldL\,[\mathsf{I}_\infty, min]$, where $min : \mathbb{Z} \times \mathbb{Z} \to \mathbb{Z}; (x, y) \mapsto x \text{ } if \text{ } x \leq y \text{ } else \text{ } y$, and $min(x, \infty) = x$ for every x.

largest The largest number of a list: $foldL\,[\mathsf{I}_{-\infty}, max]$, where $max : (x, y) \mapsto x \text{ } if \text{ } x \geq y \text{ } else \text{ } y$, and $max(x, -\infty) = x$ for every x. Here, we assume the set of integers $\mathbb{Z}$. In the case of $\mathbb{N}$, $-\infty$ can be replaced with 0.

any Determining whether any proposition is true: $any : ps \mapsto foldL\,[\mathsf{I}_{False}, \vee]\,(ps)$, where $\vee$ is logical disjunction, and ps is a list of propositions.

every Determining that all propositions are true: $every : ps \mapsto foldL[\mathsf{I}_{True}, \wedge]\,(ps)$, where $\wedge$ is logical conjunction.

reverse Reversing a list of items: $reverse : xs \mapsto foldL\,[\mathsf{I}_{[\,]}, rev]\,(xs)$, where $rev : (x, xs) \mapsto xs \oplus [x]$, and $\oplus$ appends (concatenates) two lists.

concatenate Concatenating lists of items into a single list: $concat : [xs] \mapsto foldL\,[\mathsf{I}_{[\,]}, \oplus]\,([xs])$, where $[xs]$ is a list of lists.

filter Filtering a list to retain only those items that satisfy some criterion: $filter\ f : xs \mapsto foldL\ f'\ [\,]\ (xs)$, where $f : A \to Bool; a \mapsto True \text{ } if \text{ } f(a), a \mapsto False \text{ } if \text{ } \neg f(a)$ is the criterion, and $f' : (x, xs) \mapsto x \oplus xs \text{ } if \text{ } f(x), (x, xs) \mapsto xs \text{ } if \text{ } \neg f(x)$.

For further examples, see [7].

Trees

For computation with regard to trees, we start with the polynomial functor $F_A = K_A + I \times I : \mathbf{CPO} \to \mathbf{CPO}; S \mapsto A + S \times S, f \mapsto 1_A + f \times f$. The F-algebras for the category $\mathbf{Alg}(F_A)$ are the pairs $(S, [f, g])$, where S is a set, $[f, g] : A + S \times S \to S$, $f : A \to S$ is a unary function, and $g : S \times S \to S$ is a binary function. An initial algebra in this category is $(T, [\mathit{leaf}, \mathit{branch}])$, where T is the set of trees of type A, $[\mathit{leaf}, \mathit{branch}] : A + T \times T \to T$, $\mathit{leaf} : A \to T; a \mapsto \langle a \rangle$ returns a tree consisting of a single leaf $a \in A$, and $\mathit{branch} : T \times T \to T; (l, r) \mapsto \langle l, r \rangle$ returns a tree consisting of a left branch l and a right branch r, where $l, r \in T$. That is, initial algebra $(T, [\mathit{leaf}, \mathit{branch}])$ is an F-algebra $(S, [f, g])$, where S is the set of all trees T, f is the unary function *leaf*, and g is the binary function *branch*. The tuple notation for trees is recursive, e.g., $\langle\langle a_1 \rangle, \langle\langle a_2 \rangle, \langle a_3 \rangle\rangle\rangle$ is a tree whose left branch is the leaf a_1, and whose right branch is the (sub)tree whose left branch is the leaf a_2 and right branch is the leaf a_3, depicted as

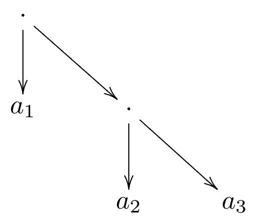

(20)

A catamorphism from initial algebra $(T, [\mathit{leaf}, \mathit{branch}])$ to an arbitrary F-algebra $(S, [f, g])$ in $\mathbf{Alg}(F_A)$ is the recursive function *foldT* (i.e., fold for trees), defined as follows. The (higher-order) function *foldT* takes a unary function $f : A \to S$ and a binary function $g : S \times S \to S$ and returns the function $\mathit{foldT}[f, g] : T \to S$, where $\mathit{foldT}[f, g] : \langle a \rangle \mapsto f(a), \langle l, r \rangle \mapsto g(\mathit{foldT}[f, g](l), \mathit{foldT}[f, g](r))$, as indicated in commutative diagram

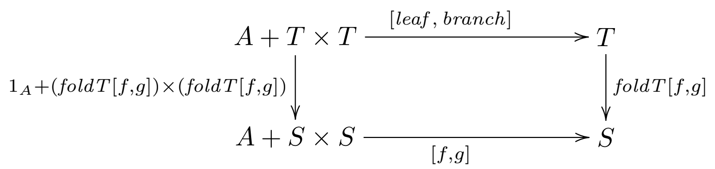

(21)

Proposition. If $f : A \to S$ and $g : S \times S \to S$ are arbitrary functions, T and S are sets, $\mathit{foldT}[f, g] : T \to S$ is a catamorphism.

Proof. First, we proved existence (commutativity), then uniqueness (by induction).

Existence (commutativity):

Leaf—$(1, a)$:

$$
\begin{aligned}
\curvearrowright\text{: } foldT[f,g] \circ [leaf, branch](1,a) &= foldT[f,g](\langle a \rangle) && \text{(definition of } [leaf, branch]) \\
&= f(a) && \text{(definition of } foldT[f,g]) \\
\curvearrowleft\text{: } [f,g] \circ (1_A + (foldT[f,g]) \times (foldT[f,g]))(1,a) &= [f,g](1,a) && \text{(definition of } 1_A + \dots) \\
&= f(a) && \text{(definition of } [f,g])
\end{aligned}
$$

Branch—$(2, (l, r))$: abbreviated to $(-)$

$$
\begin{aligned}
\curvearrowright\text{: } foldT[f,g] \circ [leaf, branch](-) &= foldT[f,g](\langle l, r \rangle) && \text{(def. of } [leaf, branch]) \\
&= g(foldT[f,g](l), foldT[f,g](r)) && \text{(def. of } foldT[f,g]) \\
\curvearrowleft\text{: } [f,g] \circ (\cdots \times foldT[f,g])(-) &= [f,g](2, (foldT[f,g](l), foldT[f,g](r))) && \text{(def. of } 1_A + \dots) \\
&= g(foldT[f,g](l), foldT[f,g](r)) && \text{(def. of } [f,g])
\end{aligned}
$$

Uniqueness: Suppose we have an F-algebra homomorphism $h : T \to S$, indicated by the commutative diagram

$$
\begin{array}{ccc}
A + T \times T & \xrightarrow{[leaf,\, branch]} & T \\
{\scriptstyle 1_A + h \times h}\downarrow & & \downarrow{\scriptstyle h} \\
A + S \times S & \xrightarrow[{[f,g]}]{} & S
\end{array}
\tag{22}
$$

Base—$(1, a)$: $\curvearrowright$: $h \circ [leaf, branch](1, a) = h(\langle a \rangle)$. $\curvearrowleft$: $[f, g] \circ (1_A + h \times h)(1, a) = [f, g](1, a) = f(a)$. Since h is an F-algebra homomorphism, $h(\langle a \rangle) = f(a) = foldT[f, g](\langle a \rangle)$.

Inductive assumption—$(2, t)$: $h(t) = foldT[f, g](t)$.

Induction step, left—$(2, (\langle a \rangle, t))$:

The commutativity of Diagram 22 means that $h \circ [\mathit{leaf}, \mathit{branch}](2, (\langle a \rangle, t)) = g(h(\langle a \rangle), h(t))$. So,

$$\begin{aligned} h(\langle a \rangle, t) &= g(h(\langle a \rangle), h(t)) && \text{(reduction of both sides)} \\ &= g(\mathit{foldT}[f, g](\langle a \rangle), \mathit{foldT}[f, g](t)) && \text{(base and inductive assumption)} \\ &= \mathit{foldT}[f, g](\langle a \rangle, t) && \text{(definition of } \mathit{foldT}\text{)} \end{aligned}$$

Induction step, right—$(2, (t, \langle a \rangle))$: Swap argument roles.

Therefore, $h = \mathit{foldT}[f, g]$. □

Examples

Computing the sum of the numbers in the leaves of a tree of numbers is the catamorphism given in commutative diagram

$$\begin{array}{ccc} \mathbb{N} + T \times T & \xrightarrow{[\mathit{leaf}, \mathit{branch}]} & T \\ {\scriptstyle 1_A + \mathit{foldT}\,[1_{\mathbb{N}},(+)] \times \mathit{foldT}\,[1_{\mathbb{N}},(+)]} \downarrow & & \downarrow {\scriptstyle \mathit{foldT}\,[1_{\mathbb{N}},(+)]} \\ \mathbb{N} + \mathbb{N} \times \mathbb{N} & \xrightarrow[{[1_{\mathbb{N}},(+)]}]{} & \mathbb{N} \end{array} \tag{23}$$

For instance, $\mathit{foldT}[1_{\mathbb{N}}, (+)](\langle\langle 1 \rangle, \langle 2, 3 \rangle\rangle) = +(1, +(2, 3)) = 6$.

Computing tree depth (i.e., the number of branches from the root of the tree to the “deepest” leaf) is the catamorphism given in commutative diagram

$$\begin{array}{ccc} A + T \times T & \xrightarrow{[\mathit{leaf}, \mathit{branch}]} & T \\ {\scriptstyle 1_A + \mathit{foldT}\,[\mathsf{1}_1,(\mathit{succ} \circ \mathit{max})] \times \mathit{foldT}\,[\mathsf{1}_1,(\mathit{succ} \circ \mathit{max})]} \downarrow & & \downarrow {\scriptstyle \mathit{foldT}\,[\mathsf{1}_1,(\mathit{succ} \circ \mathit{max})]} \\ A + \mathbb{N} \times \mathbb{N} & \xrightarrow[{[\mathsf{1}_1,(\mathit{succ} \circ \mathit{max})]}]{} & \mathbb{N} \end{array} \tag{24}$$

where $\mathit{max} : (x, y) \mapsto x$ if $x \geq y, (x, y) \mapsto y$ if $x < y$. For instance, $\mathit{foldt}(1_1, (\mathit{succ} \circ \mathit{max}))(\langle\langle 1 \rangle, \langle 2, 3 \rangle\rangle) = \mathit{succ} \circ \mathit{max}(1, 1) = 2$.

Counting the number of leaves in a tree is the catamorphism given in commutative diagram

$$\begin{array}{ccc} A + T \times T & \xrightarrow{[\mathit{leaf},\,\mathit{branch}]} & T \\ {\scriptstyle 1_A + (\mathit{foldT}\,[\mathsf{I}_1\,(+)] \times \mathit{foldT}\,[\mathsf{I}_1,(+)]}\downarrow & & \downarrow{\scriptstyle \mathit{foldT}\,[\mathsf{I}_1,(+)]} \\ A + \mathbb{N} \times \mathbb{N} & \xrightarrow[{[\mathsf{I}_1,(+)]}]{} & \mathbb{N} \end{array} \tag{25}$$

For instance, $\mathit{foldT}[\mathsf{I}_1, (+)](\langle\langle 1\rangle, \langle 2, 3\rangle\rangle) = +(1, +(1, 1)) = 3$.

Relation between numbers, lists, and trees

Under certain circumstances, operations pertaining to numbers are special cases of operations pertaining to lists, and list operations are special cases of operations pertaining to trees. The precise relationship between these domains is revealed by a category theory treatment of recursion.

Numbers from lists

For the category $\mathbf{Alg}(F)$ on the functor $F : \mathbf{CPO} \to \mathbf{CPO}; S \mapsto 1 + S, f \mapsto 1 + f$, and an arbitrary element $a \in A$, where A is a non-empty set in **CPO**, there is an initial algebra $(L, [\mathit{empty}, \mathit{cons}_a])$, where L is the set of lists constructed solely of the element a, $\mathit{empty} : 1 \to L$ is a function that returns the empty list (in effect, it is a constant), and $\mathit{cons}_a : L \to L; l \mapsto a \cdot l$ is a unary function returning the list with constant element a prepended to l. Hence, L is a set of lists where each list consists of zero or more repetitions of the element a. The catamorphism associated with this initial algebra is the function $\mathit{folda}[\mathsf{I}_v, f] : L \mapsto S; [\,] \mapsto v, (a \cdot l) \mapsto f(\mathit{folda}[\mathsf{I}_v, f](l))$, as indicated in commutative diagram

$$\begin{array}{ccc} 1 + L & \xrightarrow{[\mathit{empty},\,\mathit{cons}_a]} & L \\ {\scriptstyle 1_1 + \mathit{folda}\,[\mathsf{I}_v,f]}\downarrow & & \downarrow{\scriptstyle \mathit{folda}\,[\mathsf{I}_v,f]} \\ 1 + S & \xrightarrow[{[\mathsf{I}_v,f]}]{} & S \end{array} \tag{26}$$

The proof that $\mathit{folda}[\mathsf{I}_v, f]$ is a catamorphism follows the same pattern for *foldN*, *foldL*, etc. Since $(L, [\mathit{empty}, \mathit{cons}_a])$ is an initial algebra, we have the following corollary.

Corollary. $(L, [\mathit{empty}, \mathit{cons}_a]) \cong (N, [\mathit{zero}, \mathit{succ}])$.

Proof. Initial objects are isomorphic. □

The isomorphic relationship between $(L, [\mathit{empty}, \mathit{cons}_a])$ and $(N, [\mathit{empty}, \mathit{cons}])$, indicated in the following commutative diagram:

$$\begin{array}{ccc} 1 + L & \xrightarrow{[\mathit{empty},\, \mathit{cons}_a]} & L \\ {\scriptstyle 1_1 + \mathit{foldn}\, [\mathit{empty}, \mathit{cons}_a]} \uparrow \downarrow {\scriptstyle 1_1 + \mathit{folda}\, [\mathit{zero}, \mathit{succ}]} & & {\scriptstyle \mathit{foldn}\, [\mathit{empty}, \mathit{cons}_a]} \uparrow \downarrow {\scriptstyle \mathit{folda}\, [\mathit{zero}, \mathit{succ}]} \\ 1 + N & \xrightarrow[{[\mathit{zero}, \mathit{succ}]}]{} & N \end{array} \tag{27}$$

yields examples of primitive forms of counting and repetition.

An example of the close relationship between number and repetition follows. A list of three repeated items can be counted using *folda*: $folda\, [\mathit{zero}, \mathit{succ}]\, ([a, a, a]) = \mathit{succ}(\mathit{succ}(\mathit{succ}(\mathit{Zero})))$, which corresponds to 3. Three repetitions can be generated using *foldn*: $foldn\, [\mathit{empty}, \mathit{cons}_a]\, (\mathit{succ}(\mathit{succ}(\mathit{succ}(\mathit{Zero})))) = \mathit{cons}_a(\mathit{cons}_a(\mathit{cons}_a([\,]))) = [a, a, a]$.

F-algebras are constructed from functors, and functors are compared by natural transformations. Thus, to see the relationship between various F-algebras, we first view them as natural transformations. In general, for F-algebras on an endofunctor $F : \mathbf{C} \to \mathbf{C}$, if we have a family $\{\alpha_A : F(A) \to A\}$ of $\mathbf{C}$ – morphisms, then for any F-algebra homomorphism $f : A \to B$, the definition of F-algebra homomorphism implies that Diagram 28 commutes. That is, α_A is a natural transformation, as indicated by commutative diagram

$$\begin{array}{ccccc} A & \quad & F(A) & \xrightarrow{\alpha_A} & A \\ {\scriptstyle f}\downarrow & & {\scriptstyle F(f)}\downarrow & & \downarrow{\scriptstyle f} \\ B & & F(B) & \xrightarrow[\alpha_B]{} & B \end{array} \tag{28}$$

To compare initial algebras associated with number and with list we must use a common framework. For number, this means using an initial algebra built from the same object L used for lists, i.e. $(L, [\mathit{empty}, \mathit{cons}_a]) \cong (\mathbb{N}, [\mathit{zero}, \mathit{succ}])$. From this common framework, we see that the relationship

between initial algebras and catamorphisms is indicated by commutative diagram

$$\begin{array}{ccccc} & & \overset{[empty, cons_a \circ p_2]}{\frown} & & \\ 1 + A \times L & \xrightarrow{1_1 + p_2} & 1 + L & \xrightarrow{[empty, cons_a]} & L \\ {\scriptstyle 1_1 + 1_A \times folda[\mathsf{l}_v, f]} \big\downarrow & & {\scriptstyle 1_1 + folda[\mathsf{l}_v, f]} \big\downarrow & & \big\downarrow {\scriptstyle folda[\mathsf{l}_v, f]} \\ 1 + A \times S & \xrightarrow[1_1 + p_2]{} & 1 + S & \xrightarrow[{[\mathsf{l}_v, f]}]{} & S \\ & & \underset{[\mathsf{l}_v, f \circ p_2]}{\smile} & & \end{array} \qquad (29)$$

Recall that $p_2 : A \times L \to L$ is the projection onto the second factor of the product. Then, from the uniqueness property of catamorphisms, we have $folda[\mathsf{l}_v, f] = foldL[\mathsf{l}_v, f \circ p_2]$. Hence, every catamorphism in number is a catamorphism in list.

Lists from trees

A list can also be represented as a special kind of tree: i.e., a tree with either left-only, or right-only branches. To represent lists as trees, the formulation for a tree must be modified to yield the equivalent of lists as a special case: essentially, we use a tree where the values are embedded at the roots, instead of the leaves. This modification is straightforward, and achieved using the polynomial functor $F_A = K_1 + K_A \times I \times I : \mathbf{CPO} \to \mathbf{CPO}; S \mapsto 1 + A \times S \times S, f \mapsto 1_1 + 1_A \times f \times f$. (Compare the earlier version of trees, which used a different polynomial functor, also called $F_A = K_A + I \times I$. Note the inclusion of a terminal object to model empty lists.) The associated initial algebra for the category of F-algebras on this functor, and catamorphism are given in commutative diagram

$$\begin{array}{ccc} 1 + A \times T \times T & \xrightarrow{[empty,\, branch]} & T \\ {\scriptstyle 1_1 + 1_A \times foldT\,[\mathsf{l}_v, f] \times foldT\,[\mathsf{l}_v, f]} \big\downarrow & & \big\downarrow {\scriptstyle foldT\,[\mathsf{l}_v, f]} \\ 1 + A \times S \times S & \xrightarrow[{[\mathsf{l}_v, f]}]{} & S \end{array} \qquad (30)$$

where:

- $empty : 1 \to T$ returns an empty tree $\langle\,\rangle$;
- $branch : A \times T \times T \to T; (a, l, r) \mapsto \langle a, l, r \rangle$ returns a tree with root value $a \in A$, left branch $l \in T$ and right branch $r \in T$;

- $foldT\,[\mathsf{l}_v, f] : T \to S; \langle\,\rangle \mapsto v, \langle a, l, r\rangle \mapsto f(a, l, r)$;
- $\mathsf{l}_v : 1 \to S$ returns constant $v \in S$; and
- $f : A \times S \times S \to S; (a, s_l, s_r) \mapsto s$ is a ternary function.

Such trees contain values at their branches, depicted as follows:

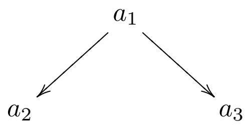

(31)

The corresponding catamorphism for counting leaves, which includes counting left/right-only list-like trees is $foldT(1_0, f)$, where $f : A \times \mathbb{N} \times \mathbb{N} \to \mathbb{N}; (a, n_l, n_r) \mapsto 1 + n_l + n_r$. For instance, the list $[John, Mary, Sue]$ is represented by the left-only branching tree $\langle John, \langle Mary, \langle Sue, \langle\,\rangle, \langle\,\rangle\rangle, \langle\,\rangle\rangle$. The number of items in this tree (list) is counted as $foldT[\mathsf{l}_0, f](\langle John, \langle Mary, \langle Sue, \langle\,\rangle, \langle\,\rangle\rangle, \langle\,\rangle\rangle) = (1 + (1 + (1 + 0 + 0) + 0) + 0) = 3$.

The right branch of a left-only branching tree is always empty, so the morphism (ternary function) *branch* simplifies to the morphism (binary function) $branchl : A \times T \to T; (a, l) \mapsto \langle a, l, \langle\,\rangle\rangle$. (An analogous simplification also applies to right-only branching trees, i.e., $branchr : (a, r) \mapsto \langle a, \langle\,\rangle, r\rangle$.) This morphism is the significant constituent of an initial algebra $(T, [empty, branchl])$, indicated in commutative diagram

$$\begin{array}{ccc} 1 + A \times T & \xrightarrow{[empty,\, branchl]} & T \\ {\scriptstyle 1_1 + 1_A \times foldtl[\mathsf{l}_v, f]} \downarrow & & \downarrow {\scriptstyle foldt[\mathsf{l}_v, f]} \\ 1 + A \times S & \xrightarrow[{[\mathsf{l}_v, f]}]{} & S \end{array} \qquad (32)$$

where $foldT\,[\mathsf{l}_v, f] : T \to S; \langle\,\rangle \mapsto v, \langle a, l, \langle\,\rangle\rangle \mapsto f(a, l)$, where $f : A \times S \to S$ is a function. Essentially, the right branch of every tree is ignored. Since $(T, [empty, branchl])$ is also an initial algebra, we have $(T, [empty, branchl]) \cong (L, [empty, cons])$.

Just like the comparison of numbers and lists, to compare initial algebras for lists and trees, we require $(T, [empty, branchl]$ as a common framework. The relationship between tree and list algebras is given by

commutative diagram

$$
\begin{array}{ccccc}
 & & \xrightarrow{[\mathit{empty}, \mathit{branchl} \circ (p_1 \times p_2)]} & & \\
1 + A \times T \times T & \xrightarrow{1_1 + (p_1 \times p_2)} & 1 + A \times T & \xrightarrow{[\mathit{empty}, \mathit{branchl}]} & T \\
\downarrow{\scriptstyle 1_1 + 1_A \times \mathit{foldtl}\, [\mathsf{I}_v, f] \times \mathit{foldTl}\, [\mathsf{I}_v, f]} & & \downarrow{\scriptstyle 1_1 + 1_A \times \mathit{foldTl}\, [\mathsf{I}_v, f]} & & \downarrow{\scriptstyle \mathit{foldTl}\, [\mathsf{I}_v, f]} \\
1 + A \times S \times S & \xrightarrow[1_1 + (p_1 \times p_2)]{} & 1 + A \times S & \xrightarrow[{[\mathsf{I}_v, f]}]{} & S \\
 & & \xrightarrow[{[\mathsf{I}_v, f \circ (p_1 \times p_2)]}]{} & &
\end{array}
\tag{33}
$$

Hence, every catamorphism in list is a catamorphism in tree.

Numbers from trees

Having shown that the initial algebras for numbers and lists, and lists and trees are related by natural transformations, the initial algebras for numbers and trees must also be related by natural transformations, since natural transformations are morphisms in a functor category, and so natural transformations compose as morphisms compose. The initial algebra for number from trees is $(T, [\mathit{empty}, \mathit{branchl}_a])$, where $\mathit{branchl}_a : T \to T; t \mapsto \langle a, t, \langle\, \rangle \rangle$. In this case, items are added (prepended) to the root of the tree.

No natural equivalences

Recall that two functors are related by a natural equivalence whenever every member of a family of morphisms that constitute a natural transformation is an isomorphism. Notice that there is also a natural transformation from the endofunctor for number, $F : 1 + S, 1_1 + f$ to the endofunctor for list, $F_A : 1 + a \times S, 1_1 + 1_A \times f$, that is indicated in the following commutative diagram

$$
\begin{array}{ccc}
1 + L & \xrightarrow{1_1 + \langle 1_1, 1_L \rangle} & 1 + A \times L \\
\downarrow{\scriptstyle 1_1 + f} & & \downarrow{\scriptstyle 1_1 + 1_A \times f} \\
1 + S & \xrightarrow[1_1 + \langle 1_1, 1_S \rangle]{} & 1 + A \times S
\end{array}
\tag{34}
$$

However, in general, the composition of these arrows with the ones in the reverse direction is not an identity, e.g., $(1_1 + p_2) \circ (1_1 + \langle 1_1, 1_S \rangle) \neq 1_{1+A\times S}$: essentially, the information regarding object A is lost. The same situation applies to the relationships between the other pairs of endofunctors. Therefore, these

endofunctors are not related by natural equivalences.

F-coalgebras, final coalgebras, and anamorphisms

F-coalgebra, final coalgebra, and anamorphism are (respectively) dual to F-algebra, initial algebra, and catamorphism. That is, their definitions are obtained by reversing the direction of the arrows in the dual construct. Here, we list the definitions.

Definition (F-coalgebra). For an endofunctor $F : \mathbf{C} \to \mathbf{C}$, an *$F$-coalgebra* is a pair (A, α), where A is an object and $\alpha : A \to F(A)$ is a morphism in $\mathbf{C}$.

Example (F-coalgebra). Let $\mathbf{C}$ be the category $\mathbf{Set}$, $F : \mathbf{Set} \to \mathbf{Set}$ be the functor $F : S \mapsto S \times S$, $f \mapsto f \times f$ (where $f : S \to T$ is a function), A be the set of natural numbers $\mathbb{N}$ (so, $\mathbb{N} \in |\mathbf{Set}|$), and $\alpha : \mathbb{N} \to \mathbb{N}$ be the morphism $\alpha : n \mapsto (p, q)$, where p is the largest prime factor of n, and $n = pq$. Then, $(\mathbb{N}, \alpha)$ is an F-coalgebra.

Definition (F-coalgebra homomorphism). An *F-coalgebra homomorphism* $h : (B, \beta) \to (A, \alpha)$ is a morphism $h : B \to A$ (in $\mathbf{C}$) such that the following diagram commutes:

$$\begin{array}{ccc} B & \xrightarrow{\beta} & F(B) \\ {\scriptstyle h}\downarrow & & \downarrow{\scriptstyle F(h)} \\ A & \xrightarrow[\alpha]{} & F(A) \end{array} \qquad (35)$$

Definition (Category of F-coalgebras). For an endofunctor $F : \mathbf{C} \to \mathbf{C}$, the category of F-coalgebras $\mathbf{CoAlg}(F)$ has F-coalgebras (A, α) for objects, and F-coalgebra homomorphisms $h : (B, \beta) \to (A, \alpha)$ for morphisms.

Definition (Final coalgebra). A *final F-coalgebra* (A, fin), hereafter also simply called a *final algebra*, is a final (i.e., terminal) object in the category of F-coalgebras $\mathbf{CoAlg}(F)$. That is, there exists a unique F-coalgebra homomorphism from every F-coalgebra in $\mathbf{CoAlg}(F)$ to (A, fin).

Like initial algebras, the presence of a final coalgebra in a category $\mathbf{CoAlg}(F)$ depends on the particular functor F, and is not guaranteed to exist in every case.

Proposition. If $F : \mathbf{C} \to \mathbf{C}$ is a polynomial functor, then the category of F-coalgebras $\mathbf{CoAlg}(F)$ has a final coalgebra.

Proof. See [3], chapters 10 and 11.

Definition (Anamorphism). An *anamorphism* $h : (B, \beta) \to (A, \mathit{fin})$ is the unique F-coalgebra homomorphism from F-algebra (B, β) to final F-coalgebra (A, fin). That is, $F(h) \circ \beta = \mathit{fin} \circ h$ and the uniquely specified h for each such β is denoted ana β (i.e., $h = \text{ana } \beta$), as indicated in the following diagram:

$$\begin{array}{ccc} B & \xrightarrow{\beta} & F(B) \\ {\scriptstyle \text{ana } \beta}\downarrow & & \downarrow{\scriptstyle F(\text{ana } \beta)} \\ A & \xrightarrow[\mathit{fin}]{} & F(A) \end{array} \tag{36}$$

where ana β is also denoted $[\!(\beta)\!]$ using *lens brackets* (see [4]).

A final coalgebra (A, fin) in $\mathbf{CoAlg}(F)$ on a functor $F : \mathbf{C} \to \mathbf{C}$ is an example of a universal construction. Specifically, it is an instance of a universal morphism (see Diagram 42).

Distributive categories and conditional functions

Before presenting specific coalgebras that we use in our explanation of systematicity, we need the definitions of *distributive categories* and *conditional functions* (see [5], p66–69, for further details).

Definition (Distributive categories). A *distributive category* $\mathbf{C}$ is a category with all products and coproducts such that we have natural isomorphisms

$$A \times (B + C) \cong (A \times B) + (A \times C)$$
$$A \times 0 \cong 0$$

The associated morphisms are called

$$\begin{aligned} \mathit{distr} &: A \times (B + C) \to (A \times B) + (A \times C) \\ \mathit{undistr} &: (A \times B) + (A \times C) \to A \times (B + C) \\ \mathit{null} &: A \times 0 \to 0 \\ \mathit{unnull} &: 0 \to A \times 0 \end{aligned}$$

Distributive categories may be thought of an analogous to the distributive law in arithmetic (and other mathematical structures such as rings and vector spaces).

Set is a distributive category, with, for example, $distr : (a, (1, b)) \mapsto (1, (a, b))$ and $(a, (2, c)) \mapsto (2, (a, c))$ for $a \in A, b \in B, c \in C$. **CPO** is also a distributive category.

Definition (Condition function). In a category **C** whose objects are sets (possibly with structure) with a boolean object $Bool = \{\text{True}, \text{False}\}$, a *condition function* $p : A \to Bool$ tests whether $a \in A$ satisfies condition (predicate) p, i.e. whether $p(a) = \text{True}$.

Definition (Conditional function). In a distributive category **C** whose objects are sets (possibly with structure), a *conditional function* $(p \to f, g) : A \to B + C$ is a function $p? : A \to A + A$, i.e., $p?(a) = (1, a)$ if $p(a) = \text{True}$ and $p?(a) = (2, a)$ if $p(a) = \text{False}$, where p is the underlying condition function, together with two functions $f, g : A \to B$ associated with condition $p : A \to Bool$ so that $(p \to f, g) : a \mapsto f(a)$ *if* $p(a)$ *else* $g(a)$. By definition, $(p \to f, g) = [f, g] \circ p?$, whose construction is indicated in commutative diagram

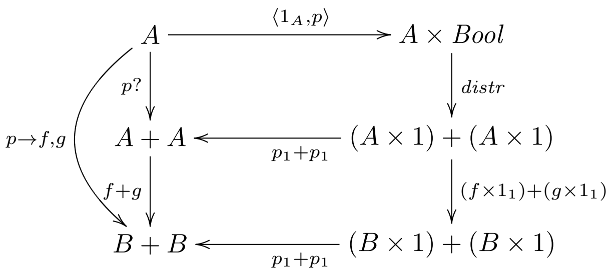

(37)

where p_1 is the usual projection onto the first factor associated with a product.

Lists

We saw how lists are constructed (put together) by initial algebras. Final coalgebras are dual to initial algebras [3]; lists are destructed (pulled apart) by final coalgebras. The resulting data structure is sometimes called a *colist* (see, e.g., [9]). Hence, the category theory development of final coalgebras for lists derives from the dual definition of initial algebra in the category of F-algebras $\mathbf{Alg}(F_A)$ on the functor $F_A : \mathbf{CPO} \to \mathbf{CPO}; S \mapsto 1 + A \times S, f \mapsto 1_1 + 1_A \times f$. That is, we start with the dual category $\mathbf{CoAlg}(F_A)$ whose objects are the F_A-coalgebras $(S, \alpha : S \to 1 + A \times S)$. An arbitrary α in this category has the form $(p \to \mathsf{l}_*, \langle f, g \rangle)$, where $\mathsf{l}_* : S \to 1$ is a constant function and $*$ is the unnamed element, $f : S \to A$, $g : S \to S$, and $p : S \to Bool$, where $p : s \mapsto \text{True}$, *if* $p(s)$ *else* False, so that $(p \to \mathsf{l}_*, \langle f, g \rangle) : s \mapsto *$, *if* $p(s)$ *else* $\langle f(s), g(s) \rangle$. That is, if $p(s)$ is true, then $\alpha(s) = (1, *)$, else $\alpha(s) = (2, (f(s), g(s)))$.

The morphisms in $\mathbf{CoAlg}(F_A)$ are F_A-coalgebra homomorphisms. A final coalgebra in this category is $(L, p_e \to \mathsf{I}_*, \langle hd, tl\rangle)$, where L is the set of lists over A, condition $p_e : L \to Bool$ tests whether $l \in L$ is empty (i.e., $l = [\,]$), and $hd : L \to A; [\,] \mapsto *, (a \cdot l) \mapsto a$ and $tl : L \to L; [\,] \mapsto [\,], (a \cdot l) \mapsto l$ return the head and tail of a list (respectively). Functions hd and tl are extended to empty lists to satisfy the general definition of a conditional function (see above) where the components are total functions. The unique F_A-coalgebra homomorphism associated with this final algebra is the anamorphism called $unfoldL'$ (i.e., unfold for lists), defined as $unfoldL'(p \to \mathsf{I}_*, \langle f, g\rangle) : S \to L$

$$
\begin{array}{ll}
s \mapsto [\,] & \text{if } p(s) \\
s \mapsto f(s) \cdot unfoldL'(p \to \mathsf{I}_*, \langle f, g\rangle)\,(g(s)) & \text{otherwise}
\end{array}
$$

The final coalgebra and associated anamorphism are shown in commutative diagram

$$
\begin{array}{ccc}
S & \xrightarrow{\;p \to \mathsf{I}_*, \langle f,g\rangle\;} & 1 + A \times S \\
{\scriptstyle unfoldL'(p \to \mathsf{I}_*, \langle f,g\rangle)} \downarrow & & \downarrow {\scriptstyle 1_1 + 1_A \times unfoldL'(p \to \mathsf{I}_*, \langle f,g\rangle)} \\
L & \xrightarrow[\;p_e \to \mathsf{I}_*, \langle hd,tl\rangle\;]{} & 1 + A \times L
\end{array} \tag{38}
$$

To show that $unfoldL'(p \to \mathsf{I}_*, \langle f, g\rangle)$ is indeed an anamorphism, we employ the *approximation lemma* [7], which is used to prove coalgebraic properties (see also [10]).

Approximation lemma (Bird). The approximation lemma is based on the function *approx*. For lists, this function is defined as $approx : \mathbb{N} \times L \to L$, where:

$$
\begin{aligned}
approx((n+1), [\,]) &= [\,] \\
approx((n+1), (x \cdot xs)) &= x \cdot approx(n, xs)
\end{aligned}
$$

By case exhaustion, $approx(0, xs) = \bot$. That is, *approx* takes on the value "undefined". For two (finite, partial, or infinite) lists xs and ys the approximation lemma is given by the following equivalence:

$$xs = ys \Leftrightarrow \forall n. approx(n, xs) = approx(n, ys)$$

That is, the two lists xs and ys are the same list if and only if their applications to the function *approx*

agree for all natural numbers n.

The approximation lemma affords proofs of coalgebraic properties via induction over the natural numbers. The *generic approximation lemma* [11] does essentially the same thing for a general class of data structures, including lists and trees.

Proposition. If $(p \to \mathsf{I}_*, \langle f, g \rangle) : S \to 1 + A \times S$ is an arbitrary conditional function, then $\mathit{unfoldL}'(p \to \mathsf{I}_*, \langle f, g \rangle)$ is an anamorphism.

Proof. First we prove existence (commutativity of Diagram 38), then uniqueness (by case analysis and the approximation lemma). (See also [10,12] for another general method of proving coalgebraic properties, called *coinduction*, which is dual to induction).

Existence (commutativity):

$p(s)$:

$$\curvearrowright\!: (1_1 + 1_A \times \mathit{unfoldL}'(p \to \mathsf{I}_*, \langle f, g \rangle)) \circ (p \to \mathsf{I}_*, \langle f, g \rangle)(s)$$

$$= (1_1 + 1_A \times \mathit{unfoldL}'(p \to \mathsf{I}_*, \langle f, g \rangle))(*) \qquad \text{(definition of } p \to \mathsf{I}_*, \langle f, g \rangle\text{)}$$

$$= (1, *) \qquad \text{(definition of } 1_1 + 1_A \times \dots\text{)}$$

$$\curvearrowleft\!: (p_e \to \mathsf{I}_*, \langle hd, tl \rangle) \circ \mathit{unfoldL}'(p \to \mathsf{I}_*, \langle f, g \rangle)(s)$$

$$= (p_e \to \mathsf{I}_*, \langle hd, tl \rangle)(*) \qquad \text{(definition of } \mathit{unfoldL}'(p \to \mathsf{I}_*, \langle f, g \rangle)\text{)}$$

$$= (1, *) \qquad \text{(definition of } p_e \to \mathsf{I}_*, \langle hd, tl \rangle\text{)}$$

$\neg p(s)$:

$$
\begin{aligned}
&\curvearrowright\!:(1_1 + 1_A \times \mathit{unfoldL}'(p \to \mathsf{l}_*, \langle f, g\rangle)) \circ (p \to \mathsf{l}_*, \langle f, g\rangle)(s) \\
&\quad = (1_1 + 1_A \times \mathit{unfoldL}'(p \to \mathsf{l}_*, \langle f, g\rangle))(f(s), g(s)) && \text{(definition of } p \to \mathsf{l}_*, \langle f, g\rangle\text{)} \\
&\quad = (2, (f(s), \mathit{unfoldL}'(p \to \mathsf{l}_*, \langle f, g\rangle)(g(s)))) && \text{(definition of } 1_1 + 1_A \times \dots\text{)} \\
&\curvearrowleft\!:(p_e \to \mathsf{l}_*, \langle \mathit{hd}, \mathit{tl}\rangle) \circ \mathit{unfoldL}'(p \to \mathsf{l}_*, \langle f, g\rangle)(s) \\
&\quad = (p_e \to \mathsf{l}_*, \langle \mathit{hd}, \mathit{tl}\rangle)(f(s) \cdot \mathit{unfoldL}'(p \to \mathsf{l}_*, \langle f, g\rangle)(g(s))) && \text{(definition of } \mathit{unfoldL}'\text{)} \\
&\quad = (2, (f(s), \mathit{unfoldL}'(p \to \mathsf{l}_*, \langle f, g\rangle)(g(s)))) && \text{(definition of } p_e \to \mathsf{l}_*, \langle \mathit{hd}, \mathit{tl}\rangle\text{)}
\end{aligned}
$$

Uniqueness: We prove uniqueness by supposing the existence of another F-coalgebra homomorphism $h : S \to L$, indicated by the commutative diagram

$$
\begin{array}{ccc}
S & \xrightarrow{\;p \to \mathsf{l}_*, \langle f, g\rangle\;} & 1 + A \times S \\
{\scriptstyle h}\downarrow & & \downarrow{\scriptstyle 1_1 + 1_A \times h} \\
L & \xrightarrow[\;p_e \to \mathsf{l}_*, \langle hd, tl\rangle\;]{} & 1 + A \times L
\end{array}
\tag{39}
$$

and then showing that $h = \mathit{unfoldL}'(p \to \mathsf{l}_*, \langle f, g\rangle)$ for all $s \in S$. For the case that $p(s)$ is true, evaluation of h does not involve recursion, and equality is essentially established by the assumption that h is an F-coalgebra homomorphism. For the case that $p(s)$ is false, evaluation of h involves recursive evaluation, and equality is established by the approximation lemma. Next, we show equality for each case.

$p(s)$: For the case where $p(s) =$ True, going clockwise in Diagram 39, since $p(s) =$ True, $s \mapsto (1, \mathsf{l}_*(s)) = (1, *)$. Since Diagram 39 commutes by assumption, this means that the anticlockwise path evaluates to $(1, *)$ as well. The only way this could happen is if $p_e(h(s))$ is True, i.e. if $h(s) = [\,]$. Hence, the definition of F-coalgebra homomorphism forces $h(s) = [\,]$, if $p(s)$. So,

$$
\begin{aligned}
\curvearrowright\!: (1_1 + 1_A \times h) \circ (p \to \mathsf{l}_*, \langle f, g\rangle)(s) &= (1, *) && \text{(definitions)} \\
\curvearrowleft\!: (p_e \to \mathsf{l}_*, \langle \mathit{hd}, \mathit{tl}\rangle) \circ h(s) &= (1, *) && \text{(}h\text{ is an }F\text{-coalgebra homomorphism)}
\end{aligned}
$$

$\neg p(s)$: For the case where $p(s) = \text{False}$, we have

$$\curvearrowright: (1_1 + 1_A \times h) \circ (p \to \mathsf{I}_*, \langle f, g\rangle)(s) = (1_1 + 1_A \times h)(f(s), g(s)) \qquad \text{(definition)}$$
$$= (2, (f(s), h(g(s)))) \qquad \text{(definition)}$$
$$\curvearrowleft: (p_e \to \mathsf{I}_*, \langle hd, tl\rangle) \circ h(s) = (2, (hd(h(s)), tl(h(s)))) \qquad \text{(definition)}$$

Since h is an F-coalgebra homomorphism, so that Diagram 39 commutes, we have the following equalities for all $s \in S$, such that $\neg p(s)$:

$$hd(h(s)) = f(s)$$
$$tl(h(s)) = h(g(s))$$
$$\Rightarrow \qquad h(s) = f(s) \cdot h(g(s)) \qquad (*)$$

We need to show that $approx(n, unfoldL'(p \to \mathsf{I}_*, \langle f, g\rangle)(s)) = approx(n, h(s))$ by induction on n to use the approximation lemma.

Base—0: Immediate, since $approx(0, xs) = \bot$ for all xs.

Assumption—n: $approx(n, unfoldL'(p \to \mathsf{I}_*, \langle f, g\rangle)(s)) = approx(n, h(s))$

Induction step—$n + 1$:

$$approx((n+1), unfoldL'(p \to \mathsf{I}_*, \langle f, g\rangle)(s))$$
$$= approx((n+1), (f(s) \cdot unfoldL'(p \to \mathsf{I}_*, \langle f, g\rangle)(g(s)))) \qquad \text{(definition of } unfoldL'\text{)}$$
$$= f(s) \cdot approx(n, unfoldL'(p \to \mathsf{I}_*, \langle f, g\rangle)(g(s))) \qquad \text{(definition of } approx\text{)}$$
$$= f(s) \cdot approx(n, h(s)) \qquad \text{(by the inductive assumption)}$$
$$= approx((n+1), (f(s) \cdot h(g(s)))) \qquad \text{(definition of } approx\text{)}$$
$$= approx((n+1), h(s)) \qquad \text{(by equality *, above)}$$

Therefore, by the approximation lemma, $h = unfoldL'(p \to \mathsf{I}_*, \langle f, g\rangle)$. □

There is a corresponding anamorphism for postpend-constructed lists, $\mathit{unfoldL}(p \to \mathsf{I}_*, \langle f, g\rangle) : S \to L$, i.e., for the category of coalgebras on the functor $F_A : S \mapsto 1 + S \times A, f \mapsto 1_1 + f \times 1_A$. The definition and proof follow $\mathit{unfoldL}'$, but with the arguments associated with the product reversed.

Trees

Like lists, the development of a final coalgebra for trees derives from its dual initial algebra (see [3]). That is, as the dual of an initial algebra in the category of F-algebras $\mathbf{Alg}(F_A)$ on the functor F_A : $\mathbf{CPO} \to \mathbf{CPO}; S \mapsto A + S \times S, f \mapsto 1_A + f \times f$. A final coalgebra in this category is $(T, (p_{\langle\,\rangle} \to \mathit{fmleaf}, \mathit{fmbranch}))$, where condition $p_{\langle\,\rangle} : T \to \mathit{Bool}$ tests whether $t \in T$ is a leaf (i.e., $t = \langle a \rangle, a \in A$), $\mathit{fmleaf} : T \to A, \langle a\rangle \mapsto a$ returns the value from a leaf, and $\mathit{fmbranch} : T \to T \times T, \langle l, r\rangle \mapsto (l, r)$ return the left and right subtrees from a branch. The dual category $\mathbf{CoAlg}(F_A)$ has as objects the F_A-coalgebras $(S, (p \to f, g))$, where $f : S \to A$ and $g : S \to S \times S$, and F_A-coalgebra homomorphisms as morphisms. The anamorphism associated with this final coalgebra is called *unfoldT* (i.e., unfold for trees), defined recursively as $\mathit{unfoldT}\,(p \to f, g) : S \to T$

$$
\begin{array}{ll}
s \mapsto \langle f(s)\rangle & \text{if } p(s) \\
s \mapsto \langle \mathit{unfoldT}(p \to f, g)(p_1 \circ g(s)), \mathit{unfoldT}(p \to f, g)\,(p_2 \circ g(s)\rangle & \text{otherwise}
\end{array}
$$

The final coalgebra and associated anamorphism are indicated in commutative diagram

$$
\begin{array}{ccc}
S & \xrightarrow{p \to f, g} & A + S \times S \\
\Big\downarrow{\scriptstyle \mathit{unfoldT}(p \to f, g)} & & \Big\downarrow{\scriptstyle 1_A + \mathit{unfoldT}(p \to f, g) \times \mathit{unfoldT}(p \to f, g)} \\
T & \xrightarrow[p_{\langle\,\rangle} \to \mathit{fmleaf}, \mathit{fmbranch}]{} & A + T \times T
\end{array}
\tag{40}
$$

Proposition. If $(p \to f, g) : S \to A + S \times S$ is an arbitrary conditional function, then $\mathit{unfoldT}(p \to f, g)$ is an anamorphism.

Proof. The proof follows a similar pattern to $\mathit{unfoldL}'$. First we prove existence (commutativity of Diagram 40), then uniqueness (by case analysis and the approximation lemma, using the *approx* function for trees, see below).

Existence (commutativity):

$p(s)$:

$$
\begin{aligned}
&\curvearrowright\!:(1_A + \mathit{unfoldT}(p \to f, g) \times \mathit{unfoldT}(p \to f, g)) \circ (p \to f, g)(s) \\
&\quad = (1_A + \mathit{unfoldT}(p \to f, g) \times \mathit{unfoldT}(p \to f, g))(1, f(s)) && \text{(definition)} \\
&\quad = f(s) && \text{(definition)} \\
&\curvearrowleft\!:(p_{\langle\,\rangle} \to \mathit{fmleaf}, \mathit{fmbranch}) \circ \mathit{unfoldT}(p \to f, g)(s) \\
&\quad = (p_{\langle\,\rangle} \to \mathit{fmleaf}, \mathit{fmbranch})(\langle f(s) \rangle) && \text{(definition)} \\
&\quad = f(s) && \text{(definition)}
\end{aligned}
$$

$\neg p(s)$:

$$
\begin{aligned}
&\curvearrowright\!:(1_A + \mathit{unfoldT}(p \to f, g) \times \mathit{unfoldT}(p \to f, g)) \circ (p \to f, g)(s) \\
&\quad = (1_A + \mathit{unfoldT}(p \to f, g) \times \mathit{unfoldT}(p \to f, g))(2, g(s)) && \text{(definition)} \\
&\quad = (\mathit{unfoldT}(p \to f, g)(p_1 \circ g(s)), \mathit{unfoldT}(p \to f, g)(p_2 \circ g(s))) && \text{(definition)} \\
&\curvearrowleft\!:(p_{\langle\,\rangle} \to \mathit{fmleaf}, \mathit{fmbranch}) \circ \mathit{unfoldT}(p \to f, g)(s) \\
&\quad = (p_{\langle\,\rangle} \to \mathit{fmleaf}, \mathit{fmbranch})(\langle \mathit{unfoldT}(p \to f, g)(p_1 \circ g(s)), \\
&\qquad\qquad \mathit{unfoldT}(p \to f, g)(p_2 \circ g(s)) \rangle) && \text{(definition)} \\
&\quad = (\mathit{unfoldT}(p \to f, g)(p_1 \circ g(s)), \mathit{unfoldT}(p \to f, g)(p_2 \circ g(s))) && \text{(definition)}
\end{aligned}
$$

Uniqueness: Following the same form of reasoning as for lists, we prove uniqueness by supposing another F-coalgebra homomorphism $h = S \to T$, indicated by the commutative diagram

$$
\begin{array}{ccc}
S & \xrightarrow{\;p \to f, g\;} & A + S \times S \\
{\scriptstyle h}\big\downarrow & & \big\downarrow{\scriptstyle 1_A + h \times h} \\
T & \xrightarrow[\;p_{\langle\,\rangle} \to \mathit{fmleaf}, \mathit{fmbranch}\;]{} & A + T \times T
\end{array}
\tag{41}
$$

and then showing that $h = \mathit{unfoldT}(p \to f, g)$ for all $s \in S$. We show equality for the two cases, i.e.,

where $p(s)$ is true, and $p(s)$ is false.

$p(s)$: For the case where $p(s) =$ True, going clockwise in Diagram 41, $s \mapsto f(s)$. Since Diagram 41 commutes by assumption, the anticlockwise path evaluates to $f(s)$ too, which can only happen if $h(s) = f(s)$. So,

$$\curvearrowright\!: (1_A + h \times h) \circ (p \to f, g)(s) = f(s) \qquad \text{(definition)}$$
$$\curvearrowleft\!: (p_{\langle\,\rangle} \to \mathit{fmleaf}, \mathit{fmbranch}) \circ h(s) = f(s) \qquad (h \text{ is } F\text{-coalgebra homomorphism})$$

$\neg p(s)$: For the case where $p(s) =$ False, we have

$$\begin{aligned}\curvearrowright\!: (1_A + h \times h) \circ (p \to f, g)(s) &= (1_A + h \times h)(2, (p_1 \circ g(s), p_2 \circ g(s))) && \text{(definition)}\\ &= (2, (h \circ p_1 \circ g(s), h \circ p_2 \circ g(s))) && \text{(definition)}\\ \curvearrowleft\!: (p_{\langle\,\rangle} \to \mathit{fmleaf}, \mathit{fmbranch}) \circ h(s) &= (2, \mathit{fmbranch}(h(s))) && \text{(definition)}\end{aligned}$$

Since h is an F-coalgebra homomorphism, so that Diagram 41 commutes, we have the following equality:

$$\begin{aligned} \mathit{fmbranch}(h(s)) &= (h \circ p_1 \circ g(s), h \circ p_2 \circ g(s)) \\ \Rightarrow \qquad h(s) &= \langle h \circ p_1 \circ g(s), h \circ p_2 \circ g(s)\rangle \qquad (^{**})\end{aligned}$$

We need to show that $\mathit{approx}(n, \mathit{unfoldT}(p \to f, g)(s)) = \mathit{approx}(n, h(s))$ by induction on n. To do so, we first need to provide the function *approx* for trees, which is defined as $\mathit{approx} : \mathbb{N} \times T \to T$ where:

$$\mathit{approx}((n+1), \langle a\rangle) = \langle a\rangle$$
$$\mathit{approx}((n+1), \langle l, r\rangle) = \langle \mathit{approx}(n, l), \mathit{approx}(n, r)\rangle$$

By case exhaustion, $\mathit{approx}(0, t) = \bot$. Induction on n proceeds as follows.

Base—0: Immediate, since $\mathit{approx}(0, t) = \bot$ for all t.

Assumption—n: $\mathit{approx}(n, \mathit{unfoldT}(p \to f, g)(s)) = \mathit{approx}(n, h(s))$

Induction step—$n+1$:

$$
\begin{aligned}
& \mathit{approx}((n+1), \mathit{unfoldT}(p \to f, g)(s)) \\
&= \mathit{approx}((n+1), (\mathit{unfoldT}(p \to f, g)(p_1 \circ g(s)), \\
&\qquad \mathit{unfoldT}(p \to f, g)(p_2 \circ g(s)))) && \text{(definition of } \mathit{unfoldT}) \\
&= (\mathit{approx}(n, \mathit{unfoldT}(p \to f, g)(p_1 \circ g(s))), \\
&\qquad \mathit{approx}(n, \mathit{unfoldT}(p \to f, g)(p_2 \circ g(s)))) && \text{(definition of } \mathit{approx}) \\
&= (\mathit{approx}(n, h(p_1 \circ g(s))), \mathit{approx}(n, h(p_2 \circ g(s)))) && \text{(inductive assumption)} \\
&= \mathit{approx}((n+1), \langle h \circ p_1 \circ g(s), h \circ p_2 \circ g(s) \rangle) && \text{(definition of } \mathit{approx}) \\
&= \mathit{approx}((n+1), (h(s))) && \text{(by equality **, above)}
\end{aligned}
$$

Therefore, by the approximation lemma, $h = \mathit{unfoldT}(p \to f, g)$. □

Universal constructions

(Co)Universal morphisms

Definition (Universal morphism). Given a functor $F : \mathbf{A} \to \mathbf{C}$ and an object $Y \in |\mathbf{C}|$, a *universal morphism* from F to Y is a pair (A, ϕ) where A is an object of $\mathbf{A}$, and ϕ is a morphism in $\mathbf{C}$, such that for every object $X \in |\mathbf{A}|$ and every morphism $f : F(X) \to Y$, there exists a unique morphism $h : X \to A$, such that $\phi \circ F(h) = f$, as indicated by commutative diagram

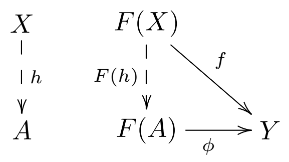

(42)

Definition (Couniversal morphism). Given an object $X \in |\mathbf{C}|$ and a functor $F : \mathbf{B} \to \mathbf{C}$, a *couniversal morphism* from X to F is a pair (B, ψ) where B is an object of $\mathbf{B}$, and ψ is a morphism in $\mathbf{C}$, such that for every object $Y \in |\mathbf{B}|$ and every morphism $f : X \to F(Y)$, there exists a unique morphism

$k : B \to Y$, such that $F(k) \circ \psi = f$, as indicated by commutative diagram

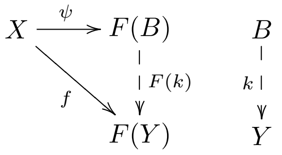

(43)

Definition (Universal construction). A *universal construction* is either a universal morphism, or (its dual) a couniversal morphism.

Adjunctions

A special kind of universal construction is called an *adjunction*, or *adjoint situation.* Our explanations for systematicity with regard to non-recursive domains employed this form of universal construction. Here, we provide definitions for comparison with initial algebras as the basis for our explanation of systematicity with regard to recursive domains.

Definition (Adjunction—unit version). An *adjunction* from categories $\mathbf{C}$ to $\mathbf{D}$ is a triple $(F, G, \eta) : \mathbf{C} \rightharpoonup \mathbf{D}$, where $F : \mathbf{C} \to \mathbf{D}$ and $G : \mathbf{D} \to \mathbf{C}$ are functors, and $\eta : 1_{\mathbf{C}} \dot{\to} G \circ F$ is a natural transformation, such that for every $X \in |\mathbf{C}|$, $(F(X), \eta_X)$ is a couniversal morphism from X to G, as indicated in the following commutative diagram:

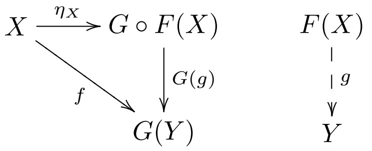

(44)

The relationship between F and G is called an *adjoint situation*, where F is the *left adjoint* of G (written $F \dashv G$), and G is the *right adjoint* of F.

Definition (Adjunction—counit version). An *adjunction* from categories $\mathbf{C}$ to $\mathbf{D}$ is a triple $(F, G, \epsilon) : \mathbf{C} \rightharpoonup \mathbf{D}$, where $F : \mathbf{C} \to \mathbf{D}$ and $G : \mathbf{D} \to \mathbf{C}$ are functors, and $\epsilon : F \circ G \dot{\to} 1_{\mathbf{D}}$ is a natural transformation, such that for every $Y \in \mathbf{D}_0$, $(G(Y), \epsilon_Y)$ is a universal morphism from F to Y, as indicated in the following

commutative diagram:

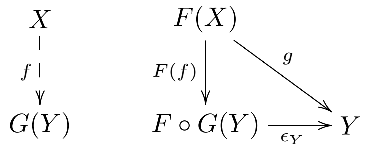

(45)

Recall that, given objects A and B in a category $\mathbf{C}$, the set $\mathbf{C}(A, B)$ is the set of all morphisms from A to B. This set is sometimes referred to as a *hom-set*, where "hom" is short for homomorphism even though a hom-set may contain morphisms that are not specifically homomorphisms.

Definition (Adjunction—hom-set version). An *adjunction* from categories $\mathbf{C}$ to $\mathbf{D}$ is a triple $(F, G, \psi) : \mathbf{C} \rightharpoonup \mathbf{D}$, where $F : \mathbf{C} \to \mathbf{D}$ and $G : \mathbf{D} \to \mathbf{C}$ are functors, and ψ is a natural isomorphism sending each pair of objects $X, Y \in \mathbf{C}^{\mathbf{op}} \times \mathbf{D}$ to a bijection of hom-sets $\mathbf{D}(F(X), Y) \cong \mathbf{C}(X, G(Y))$ that is natural in X and Y.

Various universal constructions are unified as special cases of a category theory construction called a *comma category*. Hence, the relationship between the universal construction employed here to explain systematicity with respect to recursive domains, and systematicity with respect to non-recursive domains [13, 14] is made explicit and formal via comma categories. More complete details of comma categories and their relationships to various universal constructions are given in [14] (Text S2).

References

1. Mac Lane S (2000) Categories for the working mathematician. Graduate Texts in Mathematics. New York, NY: Springer, 2nd edition.

2. Awodey S (2006) Category theory. Oxford Logic Guides. New York, NY: Oxford University Press.

3. Manes EG, Arbib MA (1986) Algebraic approaches to program semantics. New York, NY: Springer-Verlag.

4. Meijer E, Fokkinga M, Paterson R (1991) Functional programming with bananas, lenses, envelopes and barbed wire, Berlin, Germany: Springer-Verlag, volume 523 of *Lecture notes in computer science*. pp. 125–144.

5. Bird R, de Moor O (1997) Algebra of programming. Harlow, England: Prentice Hall.

6. Pierce BC (1991) Basic category theory for computer scientists. Foundations of Computing. Cambridge, UK: MIT Press.

7. Bird R (1998) Introduction to functional programming using Haskell. Prentice Hall Series in Computer Science. Harlow, England: Prentice Hall, 2nd edition.

8. Jones SP, editor (2003) Haskell 98 language and libraries: the revised report. Cambridge, UK: Cambridge University Press.

9. Uustalu T, Vene V (1999) Primitive (co)recursion and course-of-value (co)iteration, categorically. Informatica 10: 5–26.

10. Gibbons J, Hutton G (2005) Proof methods for corecursive programs. Fundamenta Informaticae 66: 353–366.

11. Hutton G, Gibbons J (2001) The generic approximation lemma. Information Processing Letters 79: 197–201.

12. Jacobs B, Rutten J (1997) A tutorial on (co)algebras and (co)induction. Bulletin of the European Association for Theoretical Computer Science 62: 222–259.

13. Phillips S, Wilson WH (2010) Categorial compositionality: A category theory explanation for the systematicity of human cognition. PLoS Computational Biology 6: e1000858.

14. Phillips S, Wilson WH (2011) Categorial compositionality II: Universal constructions and a general theory of (quasi-)systematicity in human cognition. PLoS Computational Biology 7: e1002102.
