# Supplementary material for: Categorial Compositionality III: F-(co)algebras and the Systematicity of Recursive Capacities in Human Cognition
Source: PLoS One. 2012 Apr 13;7(4):e35028. doi: 10.1371/journal.pone.0035028 (PMC3325926; doi:10.1371/journal.pone.0035028)
Supplement: Text S2 — A category theory schema for testing systematicity. (PDF) [file pone.0035028.s002.pdf]

Text S2

The benefit of a formal account of systematicity is that it makes more precise claims about cognitive capacity that can be tested empirically. Although systematicity was not formally defined in the original proposal [1], a general schema for systematicity nonetheless helps identifies cases: systematicity is evident where one has cognitive capacity c_1 *if and only if* one has cognitive capacity c_2 [1,2]. Here, we show how our category theory explanation for systematicity in terms of universal constructions can be converted into a schema for making empirically testable predictions. We specify the general form of our category theory schema for systematicity, then illustrate the idea with examples of (non-)recursive capacities.

A category theory schema for testing systematicity

A universal construction is either a universal morphism, or a couniversal morphism (see Text S1) in the domain of interest. By regarding the morphisms associated with a universal construction as (sub)tasks we have the following category theory schema for systematicity: if one has the capacity for subtasks $f'_1, \ldots, f'_n$, universal subtask u, and for composing at least one of the tasks f'_i with u so that the output of f'_i becomes the input of u, i.e. one has the capacity for the compound task $u \circ f'_i$ for some $i \in \{1, \ldots, n\}$, then one also has capacity for all compositions $f_j = u \circ f'_j$ i.e. for all $j \in \{1, \ldots, n\}$. In the case of having a couniversal morphism, the dual proposition is that having composition $f_i = f'_i \circ u$ for some $i \in \{1, \ldots, n\}$ implies having compositions $f_j = f'_j \circ u$ for all $j \in \{1, \ldots, n\}$. The objects, morphisms and composition operation, in this context, are akin to those in the category **Set**.

An example of an empirical test is adapted from the sort of transfer observed in *learning set* experimental paradigms, where participants are given a series of tasks, and the important measure is their change in performance across task instances (see, e.g., [3]). From a category theory perspective, each object is a set of stimulus/response states, and each morphism is a stimulus-response state map (transition). For example, suppose participants are given a stimulus-response task based on stimulus classes rather than individual stimuli. That is, participants are first given a classification task $f'_1 : A_1 \to C$, where stimulus $a \in A_1$ is to be classified as belonging to class $c \in C$. Then, they are given a stimulus class-response task $u : C \to R$, where each class $c \in C$ is associated with a response $r \in R$. This stimulus class-response task is the universal component of this paradigm. Then, they are given the stimulus-response task $f_1 = u \circ f'_1 : A_1 \to R$, where each stimulus $a \in A$ is associated with its target response

$r \in R$. Upon successful completion of this first stimulus-response task instance, participants are given a new task instance employing a different set of stimuli (A_2). Again, they are trained to perform a (second) classification task $f_2 : A_2 \to C$, i.e., using the same classes as the first task, but different class members. An empirical test of systematicity for this paradigm is correct prediction in the second stimulus-response task $f_2 = u \circ f'_2 : A_2 \to R$ for all $a \in A_2$.

A test of systematicity in recursive domains is to employ variations on simple counting. For example, participants are presented with a novel counting system, essentially a base four system, consisting of a set of four arbitrary symbols $A = \{j, p, g, d\}$ (e.g., shapes circle, square, triangle, diamond), and a successor function $s_A : A \to A; j \mapsto p, p \mapsto g, g \mapsto d, d \mapsto j$. First, participants are trained to recognize the correct order of symbols (i.e., the successor function). Then, participants are required to perform simple counting, e.g., count four symbols from d, i.e., produce the list $[d, j, p, g]$. Upon successful completion of this task instance, participants are given a second task instance using a different set of symbols $B = \{q, m, t, k\}$ and successor function $s_B : B \to B; q \mapsto m, m \mapsto t, t \mapsto k, k \mapsto q$. An empirical test of systematicity for this recursive domain is whether participants are able to perform simple counting over the new symbols without further training. Note that the number of symbols in each set is not required to be the same, since the couniversal morphism $[zero, succ] : 1 + \mathbb{N} \to \mathbb{N}$ that is common to both systems does not depend on set size.

The general form for testing systematicity over recursive domains is essentially the same as described for non-recursive domains: that is, participants are trained to criterion on the unique components f'_i, the common universal component u, and an instance of the target group of capacities, e.g., $f_1 = f'_1 \circ u$ (couniversal morphism case), then tested on the other instances $f_j = f'_j \circ u$ for which successful performance indicates systematicity.

References

1. Fodor JA, Pylyshyn ZW (1988) Connectionism and cognitive architecture: A critical analysis. Cognition 28: 3–71.

2. McLaughlin BP (2009) Systematicity redux. Synthese 170: 251–274.

3. Kendler TS (1995) Levels of cognitive development. Mahwah, NJ: Lawrence Erlbaum Associates.
